# Circadian rhythm disorders in patients with advanced cancer: a scoping review

**DOI:** 10.3389/fonc.2023.1240284

**Published:** 2023-09-26

**Authors:** Craig Gouldthorpe, Jenny Power, Andrew Davies

**Affiliations:** ^1^School of Medicine, Trinity College Dublin, Dublin, Ireland; ^2^Academic Department of Palliative Medicine, Our Lady’s Hospice and Care Services, Dublin, Ireland; ^3^School of Medicine, University College Dublin, Dublin, Ireland

**Keywords:** cancer, circadian rhythms, symptoms, quality of life, survival

## Abstract

Circadian rhythms can be demonstrated in several biomarkers and behavioural activities, with rhythmical patterns occurring roughly over a 24-h period. Circadian disorders occur in patients with cancer and may be associated with poor clinical outcomes. This scoping review aimed to identify circadian rhythm research and reporting practices, circadian rhythm patterns, circadian rhythm disorders, and relevant associations of circadian rhythm disorders in patients with advanced cancer. Studies involved adult patients with locally advanced or metastatic cancer and used objective measures of circadian rhythmicity. Two independent authors completed initial screening of title and abstracts, full text reviews, data extraction, and data checking. A total of 98 articles were highlighted in the scoping review, which utilised physical activity measures (actigraphy and polysomnography), biomarkers (cortisol and melatonin), or a combination. Several circadian rhythms are commonly disordered amongst patients with advanced cancer and have significant implications for symptom burden, quality of life, and survival. It remains unclear which patients are most at risk of a circadian rhythm disorder. Significant heterogeneity exists in research and reporting practices. Standardising this approach may address discrepancies in the current literature and allow for research to focus on the most relevant parameters and approaches to improving circadian rhythmicity.

## Introduction

Circadian rhythms (CRs), repeating patterns approximately every 24 h, can be observed throughout the human body in behavioural activities, such as sleeping and feeding, and biochemical and hormonal changes, such as cortisol and melatonin secretion (1). CRs are coordinated by a central “pacemaker” or “clock” situated in the suprachiasmatic nuclei within the hypothalamus that attempts to synchronise internal body clocks with the 24-h light–dark cycle (1, 2). Additionally, areas within the brain and peripherally, such as endocrine organs, contain self-sustained secondary clocks (2).

In health, two well-established endocrine biomarkers of CRs are melatonin and cortisol, with levels being measurable in several samples, including serum, saliva, and urine (2–4). Serum melatonin begins to rise from around 22:00, peaking at around 04:00, before falling towards a baseline by 10:00, which persists throughout the day. Cortisol levels peak in the early morning, around 08:00, before falling during the day to a baseline at around 00:00 (2).

Physical activity demonstrates a circadian rhythm, with peak physical activity occurring around 14:00 and the most restful period centred around 03:00, although variability does exist between individuals (5). Circadian sleep and physical activity are primarily assessed using polysomnography and actigraphy (6). Although polysomnography and actigraphy are comparable, actigraphy can be applied in various settings and allows for prolonged periods of monitoring (6). Actigraphy utilises a wrist-worn device to detect physical movement during sleep and wake periods, with analysis of data producing several measures of circadian rhythmicity (6). Actigraphy is often accompanied by patient diaries, an approach supported by the American Academy of Sleep Medicine when investigating circadian rhythm sleep disorders (7). Diaries, however, can be burdensome, inaccurately completed, and subject to bias. Adult actigraphy research has focused on sleep–wake activity, particularly sleep onset–offset and the timing of activity phases. Various measures are used within research to describe the robustness of circadian rhythmicity or the timing and relationship of events over 24-h periods (see **Table 1**).

**Table 1:** Measures of circadian rhythmicity.

Circadian measure	Description
General terms	
Acrophase	The timing of peak level (8)
MESOR	The average level over 24 h (8)
Up-MESOR	The timing of switching between low and high activity (8)
Down-MESOR	The timing of switching between high and low activity (8)
Amplitude	The difference between maximum and minimum level (8)
Double amplitude	The difference between maximum and minimum levels of the cosine function (9)
Cortisol/melatonin specific	
Area under the curve	Total cortisol levels under the curve of all measurements. Larger AUC indicates circadian disruption (10)
F test	To test the zero amplitude hypothesis (11)
Diurnal slope/diurnal decline phase	Rate of decline from cortisol peak. A smaller, more negative value indicates a steeper slope. A larger β-value, closer to 0, indicates a flatter slope, abnormal peaks, or a rising level. Calculated with log-transformed cortisol values undergoing regression analyses (12–15)
Phase angle of entrainment	The timing of the peak of the first waveform relative to awakening (16)
Dim light melatonin onset (DLMO)	Timing when melatonin exceeds a threshold considering mean and standard deviations of melatonin prior to the melatonin rise on 3 days (17)
Diurnal cortisol variability	Difference in cortisol value at earliest collection time and nighttime point (morning − night)/morning (18)
Cortisol variations (VAR)	08:00 cortisol – 20:00 cortisol/08:00 cortisol (19)
Cortisol awakening response	Cortisol slope after awakening (waking and +30 min sample) (15)
CAR_i_	Cortisol 30 min after awakening – cortisol at awakening (13)
CAR_auci_	Area under the curve during first 60 min after awakening (13)
Cortisol variability	Morning cortisol – night cortisol/morning cortisol (18)
Actigraphy specific	
General activity	
Mean activity	Mean of daily activity (20)
Intradaily variability (IV)	A measure of rhythm fragmentation (21)
Interdaily stability (IS)	A measure of rhythm stability between days (21)
VL5	Mean activity value of the 5 least active hours
L5	Mean timing of the 5 least active hours (21)
VM10	Mean activity value of 10 most active hours (21)
M10	Mean timing of the 10 most active hours (21)
Relative amplitude	(VM10 – VL5)/(VM10 + VL5) (21)
R-squared	Rhythmicity coefficient of the sleep–wake cycle (8)
Bathyphase	Time of lowest activity (22)
Circadian quotient	Amplitude/MESOR (23)
Rhythm quotient	A_24HR_/(A_4_+A_8_+A_12_) (23)
Circadian function index (CFI)	A combined measure of IV, IS, and RA (21)
Dichotomy index (I<O)	Activity in bed (I) compared to activity out of bed (O) (8, 24)
Dichotomy index for nighttime restfulness	I<O percentage of activity in bed, which falls below median activity out of bed (15)
Dichotomy index for daytime sedentariness	O<I percentage of activity out of bed, which falls below median activity in bed (15)
Autocorrelation coefficient (r24)	Correlates activity at same time points between different days, considering consistency and regularity. Higher values are more stable (15, 24)
Day-night activity balance	Ratio of activity during the day and night (23)
Night-day sleep balance	Ratio of sleep during the night and day (23)
Night-day sleep duration balance	Not described (23)
Night-day longest sleep balance	Not described (23)
Night-day per cent sleep balance	Not described (23)
Total wake time (day)	Total amount of time spent awake (25)
Movement and fragmentation index	Sum of per cent of mobile minutes and immobile bouts <1 min/no. immobile bouts within a time interval (26)
Inactivity index	Not defined (27)
Rhythm index	A measure of quality and regularity of the inactive state (17)
P1-1	Probability of staying in inactive/rest state (17)
Sleep–wake activity	
Bed time (BT)/time of retiring	Time to bed and lights switched off (27, 28)
Get up time (GUT)/time of waking up	Time woke up in the morning (27, 28)
Total time in bed	Time between BT and GUT (27, 28)
Sleep onset latency	Number of minutes to fall asleep. Time between BT and sleep onset (27–29)
Latency to persistent sleep	Number of minutes to persistent sleep (27)
Wake after sleep onset	Sum of all wake periods whilst in bed OR between sleep onset and offset (26, 27)
Total sleep time	Time between bedtime and wake time (26) OR total time in bed scored as asleep (27)
Total nighttime sleep	Sum of all sleep periods whilst in bed (26)
Sleep midpoint	Midpoint of time in bed (27, 28)
Sleep motor activity (SMA)	Mean number of movements in a given epoch (28)
Diurnal motor activity (DMA)	Mean number of movements in each epoch (28)
Wake minutes	Duration of wake during sleep period (20, 27)
Wake episodes/number of awakenings in the night (NWAK)	Number of wake episodes during sleep period (29)
Mean duration of wake episodes	Mean duration of all wake episodes (27)
Long wake episodes	Number of wake episodes lasting 5 min/+ (27, 30)
Longest wake episode	Duration of the longest wake episode (27)
Sleep fragmentation index (SFI)	Number of awakenings/total sleep time in minutes (27)
Sleep efficiency	Proportion of time asleep whilst in bed (10, 27)
Short burst inactivity index	Zero activity of 1 min/zero activity of any duration (27)
Time napping and sleep minutes	Duration of sleep episodes during wake period (20)
Long sleeps	Frequency of long naps lasting 5 min/+ (30)
Sleep episodes	Number of sleep episodes during wake period (20, 30) OR number of blocks of continuous sleep epochs (27)
Mean duration of sleep episodes	Mean duration of all sleep episodes (27)
Sleep episodes 5 min/+	Number of sleep episodes whose duration lasts 5 min or more (27)
Longest sleep episode	Duration of longest sleep episode (27)
Time awake spent immobile	The percentage of time spent awake and immobile (31).
Early morning awakening	Period of wakening in the morning lasting 30 min or longer (25)
% sleep (up interval)	Per cent of time asleep between two attempted sleep periods (32)
% sleep (down interval)	Percentage of time asleep during attempted sleep time (32)

Circadian rhythmicity can alter during an individual’s lifespan and impact on health and disease. With advancing age, activity levels decline, peak activity occurs earlier, sleep becomes shorter and more fragmented, and daytime napping increases (33, 34). Circadian rhythm disorders (CRDs), where normal rhythmicity is altered, can perpetuate cancer and metabolic, neurodegenerative, psychological, and cardiovascular disease (35). CRDs are common amongst cancer patients, affecting up to 75%, and are associated with increased symptom burden, poorer quality of life, and shorter survival (36, 37). Interestingly, even misalignment between preferred and actual bedtimes is associated with cancer progression (38).

## Aims

This review will broadly consider circadian rhythms of cortisol, melatonin, and physical activity in advanced cancer patients, with the aim of:

1. Identifying investigative approaches and reported parameters
2. Identifying circadian rhythm and disordered rhythm patterns
3. Identifying associations with circadian rhythm disorders, focusing on symptoms, quality of life, and survival.

## Methodology

### Data sources

A literature search was performed using PubMed, Embase, Web of Science, Ebsco host (CINAHL, Psychinfo, and Psycharticles), Scopus, and Cochrane on 20/04/2022. The search was updated on 05/05/2023. Keywords were restricted to title and abstract. No other limitations were placed.

### Search terms

An example search strategy within PubMed is as follows: (“circadian”[Title/Abstract] OR “sleep wake”[Title/Abstract] OR “rest activity”[Title/Abstract] OR “chrono*”[Title/Abstract] OR “clock”[Title/Abstract] OR “Chronobiology Disorders”[MeSH Terms]) AND ((“advanced”[Title/Abstract] OR “progressive”[Title/Abstract] OR “palliat*”[Title/Abstract] OR “terminal”[Title/Abstract] OR “metast*”[Title/Abstract] OR “end of life”[Title/Abstract]) AND (“cancer*”[Title/Abstract] OR “malig*”[Title/Abstract] OR “tumo*”[Title/Abstract] OR “neop*”[Title/Abstract] OR “oncol*”[Title/Abstract] OR “Neoplasms”[MeSH Terms])).

### Eligibility

Studies were eligible for inclusion if the patients were ≥18 years old with a diagnosis of advanced cancer (locally advanced or metastatic). “Locally advanced” differed between cancer histology and several studies included, rather than focused solely on, patients with advanced cancer. Eligible studies also had to consider objective measures of four markers of circadian rhythm disorders (sleep–wake cycles, rest–activity cycles, cortisol levels, and melatonin levels) and be fully translated into English.

### Screening, data extraction, and data synthesis

Two authors (CG and JP) independently screened the title and abstract for potential full-text review. Review papers identified in the initial search were also screened for additional articles. Full-text articles were reviewed independently by two authors (CG and JP). The reference lists of included articles were searched for additional articles. Where full-text copies were not immediately available, the leading author or associated research centre was contacted, and if no full-text made available, the article was excluded. Data were extracted by a single author (CG) and confirmed independently by a second author (JP). The data extraction tool was then coded into main themes including circadian measures, circadian rhythm patterns, and the association of circadian measures with symptoms, quality of life, survival and other relevant factors. The review is presented according to the PRISMA-ScR checklist.

## Results

**Figure 1:**
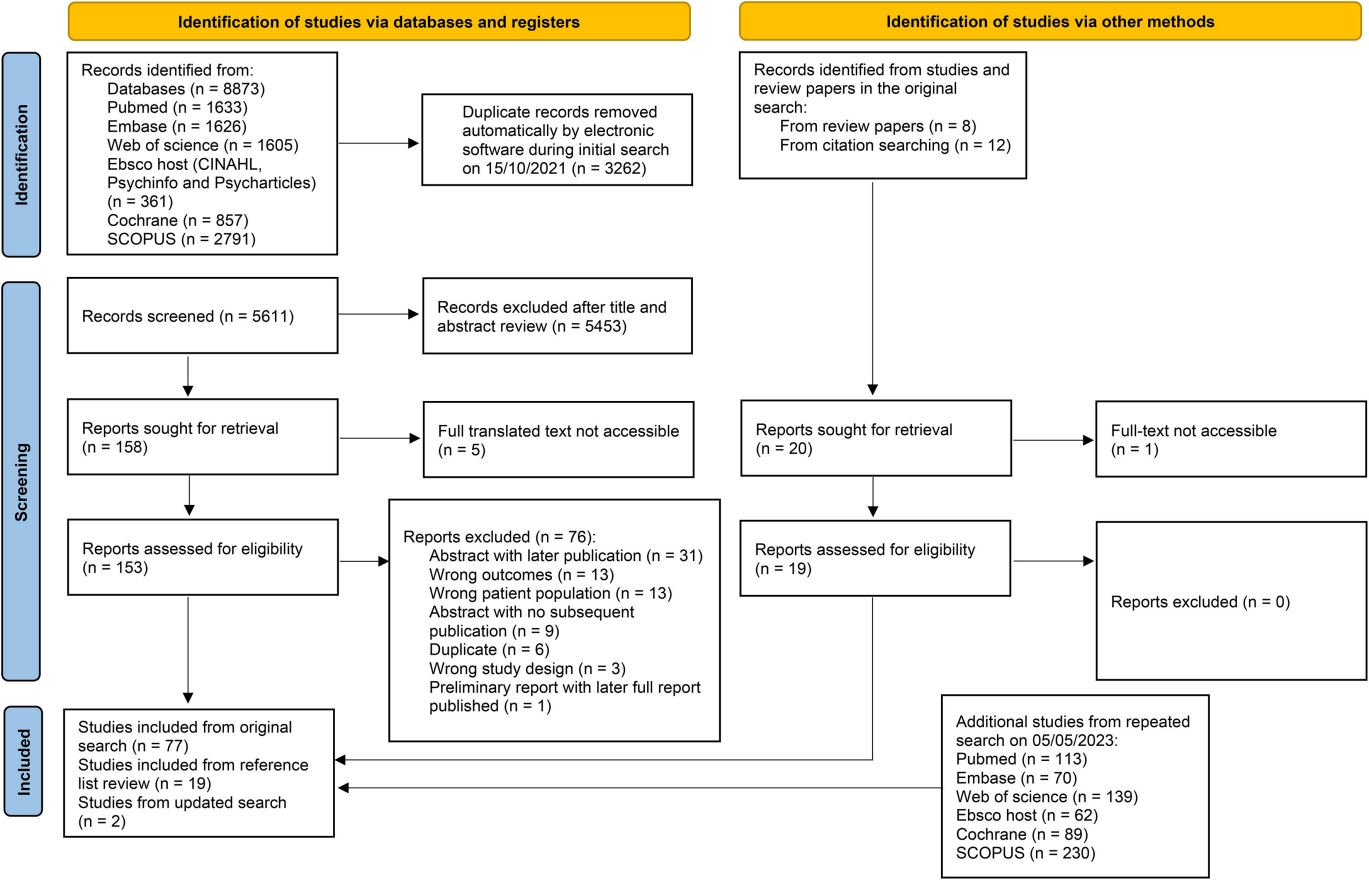
A flow chart of article identification, screening, and exclusion. *From:* Page MJ, McKenzie JE, Bossuyt PM, Boutron I, Hoffmann TC, Mulrow CD, et al. The PRISMA 2020 statement: an updated guideline for reporting systematic reviews. BMJ 2021;372:n71. doi: 10.1136/bmj.n71. For more information, visit: http://www.prisma-statement.org/.

The scoping review highlighted 98 articles, which were mainly observational in nature. The review process can be seen in **Figure 1**, and the results from individual studies are detailed in **Tables 2A–D**.

**Table 2A:** Melatonin circadian rhythms and their associations in patients with advanced cancer.

Studies with a control group			
Article	Participants	Circadian measures	Outcomes relevant to review
Levi et al., 2020 (17)	“IDEAs” study: 25 (21 M) locally advanced or metastatic gastrointestinal cancer patients, median age 66 years “PicaPill” study: 33 (15 M) control subjects, median age 35 years	6-h salivary melatonin levels (19:00, 20:00, 21:00, 22:00, 23:00, 00:00) and the dim-light melatonin onset (DLMO), which represents the time melatonin levels rise above a threshold.	Melatonin levels rose from baseline in all participants between 18:00 and 23:00. Patients with cancer had higher baseline melatonin levels. The rise in melatonin was higher in controls and patients with cancer with relatively less in-bed to out-of-bed physical activity (threefold low I<O group, fivefold high I<O group, and sixfold in control subjects). Patients with cancer and relatively less daytime to night-time activity had earlier DLMOs (1,948 vs. 2,144, p=0.08). Significant inter-individual variation was noted.
Mazzoccoli et al., 2012 (39)	9 (M) stage 2–4 non-small cell lung cancer patients, mean age 51 years 11 (M) control subjects, mean age 44 years	24-h rhythm of serum melatonin: (06:00, 10:00, 14:00, 18:00, 22:00, 02:00)	A 24-h rhythm was found in all subjects with a peak concentration at night, and a trough concentration near waking. Mean values did not differ between the groups at any time points.
Hu et al., 2009 (40)	30 (26 M) “advanced” non-small cell lung cancer patients, mean age 60 years 63 (53 M) control subjects, mean age 67 years	Serum melatonin levels and 24-h rhythm (12:00, 00:00) and urine 6-sulfatoxymelatonin levels (major metabolite of melatonin) (07:00, 16:00)	A 24-h rhythm of melatonin and 6-sulfatoxymelatonin were present in all subjects. Serum melatonin at 00:00 was lower in patients than in control subjects (p<0.05). Urine 6-sulfatoxymelatonin at 07:00 and 16:00 was lower in patients than in control subjects (p<0.05)
Karasek et al., 2005 (41)	31 (F) stage 0–4 cervical cancer patients, mean age 53 years 14 (F) control subjects, mean age 54 years	Serum melatonin levels and area under the curve (AUC) (08:00, 12:00, 16:00, 20:00, 24:00, 02:00, 04:00, 08:00)	Cancer patients had significantly lower melatonin levels and area under the curve (AUC) than control subjects (p<0.05). “Nocturnal” melatonin levels and the AUC were significantly lower in patients with stage 3–4 cancer compared to patients with stage 0–1 cancer (p<0.05).
Mazzoccoli et al., 2005 (42)	17 stage 1–2 non-small cell lung cancer patients, mean age 67 years 17 stage 3–4 non-small cell lung cancer patients, mean age 70 years 17 control subjects, mean age 69 years	24-h rhythm of serum melatonin and AUC (06:00, 10:00, 14:00, 18:00, 22:00, 02:00, 06:00)	A 24-h rhythm was present in all three groups. AUC levels were lower in cancer patients (p<0.05) and lower in cancer patients with a higher cancer stage (p=ns)
Muc-Wierzgon et al., 2003 (43)	42 (25 M) “advanced” (metastatic) gastrointestinal cancer patients, mean age 61 years 30 (25 M) control subjects, mean age 57 years	Serum melatonin levels, 24-h rhythm (08:00, 14:00, 18:00, 22:00, 02:00, 08:00), amplitude (difference between peak and trough levels) and acrophase (time of peak level)	A 24-h rhythm was noted in all subjects. The maximal peak levels were higher for control subjects, but the minimal trough levels were similar for control subjects and patients. The mean amplitude was higher for control subjects. The acrophase occurred earlier for control subjects (04:35 vs. 08:50).
Ermachenkov et al., 2013 (44)	89 (49 M) gastric cancer patients (8 metastatic) 86 (31 M) colorectal cancer patients (5 metastatic) Mean age 62 years	Diurnal urinary 6-sulfatoxymelatonin levels	Gastric cancer patients with distant metastases had lower diurnal excretion than patients without distant metastases (231±27 ng/h vs. 422±36 ng/h, p<0.001). Colorectal cancer patients with metastatic disease had lower diurnal excretion than patients without metastatic disease (176±44ng/h vs. 422±36ng/h, p<0.001).
Karasek et al., 2000 (45)	23 (F) mixed gynaecological cancer, mean age 50 years, included “invasive ovarian” 16 (F) control subjects, mean age 51 years 7 (F) myomatous uterus patients, mean age 46 years	24-hour rhythms and AUC of serum melatonin (08:00, 12:00, 16:00, 20:00, 22:00, 24:00, 02:00, 04:00, 06:00, 08:00)	There was no significant difference in the 24-h rhythm and AUC between the three groups.
Baranowski et al., 1999 (11)	30 (17 M) stage 4 gastrointestinal cancer patients, mean age 64 years 29 age-matched healthy control subjects (gender not defined)	Serum melatonin: 08:00, 14:00, 18:00, 22:00, 02:00, 08:00	A 24-h rhythm was present for all subjects. Compared to controls, patients with cancer had similar minimum levels (12.1 pg/ml vs. 12pg/ml), lower maximum levels (34.3pg/ml vs. 65pg/ml), and lower average levels (MESOR) (23.1pg/ml vs. 48.2pg/ml)
Tarquini et al., 1999 (46)	39 (17 M) mixed cancer patients (21 metastatic), mean age 72 years 28 (11 M), control subjects, mean age 65 years	24-h serum melatonin levels and amplitude (00:00, 04:00, 08:00, 12:00, 16:00, 20:00, 24:00)	A 24-h rhythm was present for all subjects. The amplitude was smaller in cancer patients than control subjects (p=0.003) with higher daytime levels and lower night-time levels. No difference in amplitude was found in relation to cancer stage.
Dogliotti et al., 1990 (47)	Study 1: 132 (90 F), stage 1–4 mixed cancer patients, median age 63 years. 58 (32 M) control subjects, median age 35 years Study 2: 20 stage 1–3 breast cancer patients, median age 60 years Study 3: 18 mixed cancer patients, age and gender not defined, Control subjects age, gender, and number not defined	Study 1: Serum melatonin (08:00, 24:00) Study 2: Serum melatonin (08:00, 24:00) Study 3: 24-h serum melatonin (08:00, 12:00, 16:00, 20:00, 00:00, 04:00, 08:00)	Study 1: Melatonin levels were higher at both time points in patients than controls (p<0.0001). Stage 4 breast cancer patients had higher mean melatonin concentration than controls (p<0.0001) and higher levels at 24:00 (p<0.002) and 08:00 (p<0.0001) than stage 1–2 breast cancer patients. Advanced lung cancer patients had higher mean melatonin levels than control at both time-points (p<0.001 at 24:00, p<0.0001 at 08:00). Highest levels were in patients with SCLC. Advanced GI cancer patients had higher mean melatonin levels than control (p<0.005 at 24:00, p<0.001 at 08:00). Increased melatonin levels at 08:00 were associated with lower performance status (r=−37, p<0.01). Study 2: Melatonin levels did not differ in breast cancer patients pre- and post-surgical removal of the primary tumour Study 3: The circadian melatonin rhythm was similar between patients and controls.
Bartsch et al., 1981 (48)	10 (F) stage 1–4 breast cancer patients, mean age 57 years 10 (F) control subjects, mean age 53 years	24-h urinary melatonin (06:00–10:00, 10:00–14:00, 14:00–18:00, 18:00–22:00, 22:00–06:00)	Cancer patients had a lower average melatonin urinary excretion and elevated levels between 06:00 and 10:00 than controls. The differences were not statistically significant. A more synchronised excretion pattern was found in controls
Studies without a control group			
Mormont et al., 2002 (49)	18 (14 M) metastatic colorectal cancer patients, mean age 58 years	24-h rhythms for serum melatonin and serum 6-alphasulfatoxymelatonin on 3–6 h apart for 10–13 times points	15/18 (83%) patients had a 24-h serum melatonin rhythm and 6/18 (33%) had a 24-h 6-alpha-sulfatoxymelatonin rhythm.
Mormont et al., 1998 (50)	18 (14 M) metastatic colorectal cancer patients, age 35–72 years	24-h rhythm of blood melatonin	A group 24-h rhythm was evident for melatonin (p<0.00001) and significant circadian melatonin rhythm for 15 patients (p<0.05). Wide interindividual variation was noted.
Studies without a control group			
Article	Participants	Circadian measures	Outcomes relevant to review
Vivani et al., 1992 (51)	7 (6 M) small cell lung cancer patients, median age 51 years Patients administered IL-2	Baseline and weekly serum melatonin (08:00, 16:00, and 24:00)	Abnormal melatonin 24-h rhythms were found in all patients at baseline which was absent in 5 patients and had an earlier acrophase in 2 patients. The mean values were not significantly higher than those at 08:00 or 16:00.

M, male; F, female; AUC, area under curve; ns, not significant.

**Table 2B:** Cortisol circadian rhythms and their associations in patients with advanced cancer.

Studies with a control group			
Article	Participants	Circadian measures	Outcomes relevant to review
Levi et al., 2020 (17)	IDEAs study: 25 (21 M) locally advanced or metastatic gastrointestinal cancer patients, median age 66 PicaPill study: 33 (15 M), control subjects, median age 35	2-day salivary cortisol (3-hourly) and 7-day wrist actigraphy and chest accelerometry and 1-day melatonin samples hourly at 19:00	A consistent diurnal change in cortisol levels was seen in most controls and patients, irrespective of their dichotomy index (I<O), which represents the relative difference between in-bed and out-of-bed physical activity. Those with a high I<O (i.e., relatively less in-bed to out-of-bed activity) had a larger circadian cortisol amplitude (difference between peak and trough concentrations). No significant difference was found in other cortisol parameters between I<O groups.
Zeitzer et al., 2016 (52)	97 (F) recurrent or metastatic breast cancer patients, age 57.6 24, health age-matched controls, age 57.1 (gender not identified)	Combination: 28-h plasma cortisol (20–60-min intervals) and polysomnography and 2-week actigraphy (Actiwatch 2) and sleep diary	There were no differences in the cortisol amplitude, MESOR (mean value) or absolute/relative timing between groups (p>0.09). There were no differences in the diurnal cortisol rhythm between groups (p>0.11). Abnormal cortisol peaks, midway through the sleep episode, were seen in a subset of patients and were associated with increased wake episodes (p=0.004), metastases to bone or organs rather than local recurrence (r=−0.37, p=0.002), use of steroids (r=0.26, p=0.03), ER negative status (r=−0.25, p=0.04) and higher a stage of initial diagnosis (r=0.31, p=0.009). In a multivariate analysis, metastases to bone (p=0.02) and ER negative status (p=0.048) continued to be associated with the abnormal cortisol peaks. Abnormal cortisol peaks were not related to psychological traits (p>0.018). Larger abnormal peaks were associated with a shorter disease-free interval (r=−0.30, p=0.004). The disease-free interval (DFI) and the diurnal cortisol rhythm were not associated (p>0.10).
Du et al., 2013 (53)	25 (15 M) stage 2–4 lung cancer patient with depression, mean age 55.1 39 (23 M) stage 2–4 lung cancer patients without depression, mean age 57.0 21 (8 M) patients with depression, mean age 53.8 41 (21 M) control subjects, mean age 55.9	24-h salivary cortisol (08:00, 16:00, 00:00, 06:00)	Lung cancer patients with depression had a flattened circadian cortisol pattern (less diurnal variation) compared to other groups. Lung cancer patients also had higher salivary cortisol at 00:00 compared to lung cancer patients without depression (p<0.001). The salivary cortisol area under the curve (AUC) was significantly higher in patients with depression only than the other groups (p = 0.021). Salivary cortisol diurnal variation (VAR) was significantly lower in lung cancer patients with depression than other groups (p<0.001).
Kim et al., 2012 (13)	52 (37 M) stage 3a–4 lung cancer patients, mean age 60.8 56 (32 M), control subjects, mean age 60.7	2-day salivary cortisol (waking, +30 min, +60 min, 21:00)	The cortisol awakening rise (CAR) represents the rapid rise in cortisol on awakening, and levels at 0, + 30 min and +60 min were significantly higher in controls than in patients (p<0.05). CARauci and CARi were higher in controls than in patients (p<0.05). Cortisol levels at 21:00 were similar between patients and controls. Flatter diurnal cortisol slopes were seen in patients compared to controls (p<0.001). Decreased cortisol levels and abnormal secretory patterns were seen in patients with an ECOG PS of 3 and 4 compared to controls (p<0.001). A positive correlation was seen between the diurnal cortisol slope and clinical disease stage (p<0.01). CARi and CARauci were not associated with clinical disease stage. For patients, gender, number of metastatic sites, chemotherapy status, body mass index, and smoking status were independent of the cortisol profile (p>0.05).
Mazzoccoli et al., 2012 (39)	9 (M) stage 2–4 non-small cell lung cancer patients, mean age 51.0 11 (M) control subjects, mean age 43.6	Combination: serum cortisol and melatonin (06:00, 10:00, 14:00, 18:00, 22:00, 02:00)	Prominent 24-h cortisol rhythms were seen in all subjects with peaks at night for melatonin and near waking for cortisol. Mean cortisol values did not differ between groups at any time points. The overall 24-h mean for cortisol was higher in cancer patients than controls (p=0.001). An increased cortisol slope was associated with increasing disease severity (p<0.001).
Weinrib et al., 2010 (54)	100 (F) stage 1–4 ovarian cancer patients, mean age 58.19 77 (F) benign disease, mean age 51.04 33 (F) control subjects, mean age 52.79	Salivary cortisol (waking, 16:00–18:30, bedtime)	Mean afternoon cortisol for ovarian cancer patients was 55% higher than for healthy women (p<0.0001) and similar to patients with benign disease (p=0.07). Nocturnal cortisol levels for ovarian cancer were 41.5% higher than benign disease (p=0.02) and 103% higher than healthy women (p=0.0001). Cortisol variability of ovarian cancer patients was lower than for benign disease (p=0.023) and healthy women (p<0.0001). Adjusted for age and disease stage in the ovarian group, a higher nocturnal cortisol, and lower cortisol variability was associated with greater fatigue (p=0.005 and p = 0.01). Lower cortisol variability also associated with poorer physical well-being (p=0.007). Depression scores were associated with a higher nocturnal cortisol (p=0.059) and lower cortisol variability (p=0.028). A more advanced cancer stage was associated with a higher morning (r=0.23, p=0.02) and afternoon (r=0.32, p=0.002) cortisol, but not nocturnal cortisol (r=0.13, p=33). Adjusted for age and disease stage in ovarian cancer group: higher nocturnal cortisol associated with poorer physician-related PS (rated on a 0–4 scale) (p=0.043) and patient-related PS (p=0.035). Lower cortisol variability was also associated with poorer physician-rated PS (p=0.01) and poorer patient-rated PS (p=0.004).
Wu et al., 2008 (55)	13 (9 M) stage 2–4 nasopharyngeal cancer, median age 41 14 (8 M) healthy control subjects, mean age 24.5	24-h plasma cortisol (4 hourly sampling)	Patients had a lower cortisol MESOR compared to control (200.31 ± 14.38 nmol/L vs. 243.77 ± 14.96 nmol/L, p=0.30) The acrophase was later for patients than controls (09:14 vs. 08:41) The amplitude was higher for patients than controls (64.01 vs. 61.94) A clear cortisol circadian rhythm with peaks in the morning was seen in both groups
Mazzoccoli et al., 2005 (42)	17 healthy subjects, mean age 68.8 17 stage 1–2 non-small cell lung cancer patients, mean age 67.2 17 stage 3–4 non-small cell lung cancer patients, mean age 69.5	Combination: 24-h serum cortisol and melatonin (4 hourly)	No significant different in cortisol levels was seen amongst groups. Cancer patients did not show a clear rhythm of cortisol secretion.
Abercrombie et al., 2004 (12)	17 (gender not defined) metastatic breast cancer patients, mean age 57.6 31 (F) control subjects, mean age 56.0	3-day salivary cortisol at waking (mean collection time, 07:17), 12:00, 17:00, and 21:00	Patients had significantly flatter diurnal cortisol slopes than controls (p<0.05). Disease severity was associated with flatter diurnal cortisol slopes (p=0.07) and higher mean cortisol levels (p<0.05). Mean cortisol levels between groups were not significantly different at different time points. Diurnal cortisol slopes and mean cortisol levels did not correlate with psychological measures (p>0.32).
Baranowski et al., 1999 (56)	30 (17 M), “advanced” gastric, pancreatic, or colorectal cancer patients, mean age 63.5 20 control subjects (gender not defined), mean age 59.5	Serum cortisol (08:00, 14:00, 18:00, 20:00, 02:00, 08:00)	A “well-defined circadian rhythm” was seen in control subjects, with a MESOR (average) of 116.8pg/ml, amplitude (difference between peak and trough) of 85.5pg/ml and acrophase (timing of peak concentration) of 07:20. In patients, a later acrophase of 08:08 at 520±11.8ng/ml was noted, and the trough occurred at 18:00 at 279.7±3.7 ng/ml.
Mormont et al., 1998 (9)	3 study cohorts 1. 19 (7 M) control subjects, 7 young (mean age, 24), 6 elderly women (mean age, 74.7), 6 elderly men (mean age, 71.7) 2. 19 (F) advanced ovarian cancer patients, mean age 59 3. 18 (14 M) advanced metastatic colorectal cancer patients, mean age 58	Serum cortisol Retrospective studies—4 circadian time series at 3-monthly intervals Prospective study—5–6 samples in 1st and 4th day of chemotherapy	The mean cortisol peak occurred at 08:00. The cortisol trough occurred earlier in controls (20:00) than in ovarian and colorectal cancer patients (00:00 or 01:00). Mean serum cortisol amplitude was 30% lower in cancer patients compared to controls (ovarian p=0.01, colorectal cancer p=0.002). There was no significant influence of age, gender, performance status, percentage of liver replacement, or number of metastatic sites on the mean estimate of circadian amplitude in the colorectal cancer patients. Ovarian cancer patients with a WHO PS 3–4 had a significantly lower MAX-MIN and lower mean H8-16 cortisol than those with a performance status of 2 or less.
Singh et al., 1998 (57)	25 (F), early (TNM B-1) and advanced (TNM B-2) breast cancer patients, aged 25–60 years 15(F), control subjects, aged 25–40 years	24-hour urine 17-ketogenic steroid (17-KGS) and 17-ketosteroid (17-KS) at 6 hourly collections 17-KGS and 17-KS are metabolites that may be derived from adrenal steroids and androgens from the gonads	A significant circadian rhythm of urinary 17-KGS in controls and early-stage breast cancer (all p<0.001) with an acrophase of 18:14 in controls and 18:55 in early-stage breast cancer. In advanced-stage breast cancer the acrophase occurred at 16:26 with elevated values at almost all time points compared to controls and early-stage breast cancer patients. A significant circadian rhythm of urinary 17-KS was noted in controls. The acrophase was 21:18. A circadian rhythm of urinary 17-KS was also found in early-stage breast cancer with an acrophase around 20:59. An irregular circadian rhythm of urinary 17-KS was noted in patients with advanced breast cancer with an acrophase of 20:16, when compared to controls and early-stage breast cancer
Payer et al., 1997 (58)	11 (8 M), bowel cancer patients (4 metastatic) median age 65 17 (13 M), ulcerative colitis patients, median age 32.5, 28 (15 M) patients with large bowel polyps, median age 32.5 13 (10 M) health controls, median age 21	Serum cortisol (08:00, 12:00, 16:00, 20:00, 04:00, 08:00)	Cancer patients had a lower cortisol amplitude (p<0.05) and shorter 12-hour acrophase (p<0.05) than other groups.
Singh et al., 1995 (59)	25 (F) early and advanced breast cancer patients, aged 25–60 15 (F) control subjects, aged 25–40	Serum 17-hydroxycorticosteroid (OHCS) (06:00, 12:00, 18:00, 00:00)	Control subjects had a mean 17-OHCS of 19.21μg/dl at 06:00, which reduced throughout the day to minimum concentrations at 00:00. A significant difference at time points was found (p<0.001). The MESOR was 13.2±0.55μg/dl, and amplitude was 5.43μg/dl. The amplitude was significantly different from zero. The acrophase occurred at 08:56. Patients with advanced breast cancer had higher 17-OHCS at 06:00 than controls (34.56μg/dl), and an earlier acrophase of 04:38. Breast cancer patients have a higher MESOR than controls (p<0.001).
Singh et al., 1987 (60)	10 (F) advanced breast cancer patients, aged 35–60 Patient group split into two groups according to their circadian pattern (not further defined) 10 (F) control subjects, aged 25–40	24-h serum cortisol at 8-hourly intervals from 08:00	Control subjects had a mean 17-OHCS at 08:00 of 18.03μg/dl with a minimum level at 00:00. The amplitude was significant from baseline (p=0.001) suggesting a marked circadian rhythm. Group 2 (6 patients) had deranged circadian rhythms with no significant difference in mean values between time points or in the change in cortisol level from baseline. Group 3 (4 patients) had a mean 17-OHCS at 08:00 of 25.20μg/dl with a minimum at 00:00. A normal circadian rhythm was seen with a significantly amplitude from baseline (p=0.05).
DeMeester et al., 1979 (61)	76 stage 1–3 non-oat-cell bronchogenic carcinoma 15 control subjects with suspected carcinoma but found to have benign disease Age and gender not defined	Serum cortisol (08:00, 16:00, 00:00)	Stage 1 patients’ cortisol levels at all time points did not significantly differ from controls. Stage 2–3 patients had higher 08:00 and 16:00 levels than control subjects and stage 1 (p<0.05). Stage 3 metastatic patients had similar 00:00 levels to 08:00 peak of controls. 66 patients maintained a normal diurnal rhythm with significantly higher 08:00 levels to 00:00. Two patients lost their diurnal variation and in 8 patients the rhythm was reversed with 00:00 levels higher than 08:00. Elevated cortisol at 00:00 was associated with progressive disease but not length of survival.
Bishop et al., 1970 (62)	80 inoperable lung cancer patients admitted for radiotherapy/chemotherapy, 45 inoperable or metastatic cancer patients (not lung) 35 control subjects admitted for minor surgery Age and gender not defined	Serum cortisol (basal 08:30–09:30, midnight 23:00–24:00, 08:30–09:30 the morning after 2mg dexamethasone)	Cancer patients had significantly higher 8 a.m. cortisol than controls. A reduction in the diurnal cortisol variation was seen in cancer patients. All cancer patients showed less cortisol suppression following dexamethasone than control. No significant correlation was found between TNF-alpha and cortisol. Significance levels not reported
Studies without a control group			
Cheung et al., 2021 (10)	30 (16 M) stage 3b-4 non-small cell lung cancer patients Group 1—Aerobic exercise, mean age 61.00 Group 2—Tai-Chi, mean age 61.11 Group 3—Self-management group, mean age 58.36	Combination: Salivary cortisol rhythms (0.5, 4, 8, and 12 h after waking) and 3-day actigraphy (AMI)	The diurnal cortisol slope (representing the decline in cortisol levels during the day following the morning peak) and the cortisol area under the curve values at baseline were identified but no correlations reported. No control group was included for comparison.
Allende et al., 2020 (63)	99 (F) metastatic or recurrent breast cancer patients, median age 54	3-day salivary cortisol at waking, +30, 12:00, 17:00, and 21:00	The diurnal cortisol slope data was split at the median point to distinguish flat and steep slopes. Flat and steep diurnal cortisol slopes had significantly different salivary cortisol levels at 12:00 (p=0.0086), 17:00 (p<0.0001), and 21:00 (p<0.0001), but not at waking (p=0.4795) or waking +30 min (p=0.1364). This suggests that the differences between flat and steep cortisol slopes occur at 12:00 or later. Flatter diurnal cortisol slopes were associated with an escape from dexamethasone suppression (p=0.0042).
Studies without a control group			
Article	Participants	Circadian measures	Outcomes relevant to review
Oh et al., 2019 (64)	46 (39 M), stage 2-4 small cell lung cancer and non-small cell lung cancer patients (15 patients had metastatic disease), 23 ≤65 years old, 23 >65 years old	24-h salivary cortisol (waking, +30 min, +60 min, 21:00–22:00)	Cortisol concentrations differed between patient and controls (p<0.001). The cortisol awakening response (CAR) represents changes in cortisol levels within given time periods. CARi (the cortisol increase in the first 30 min after waking) and CARauc (the cortisol increase in the first 60 min from waking) were both significantly smaller in the patients compared to controls (p<0.01). A flatter diurnal cortisol slope was seen in the patients compared to controls (p<0.001). Patients with Eastern Cooperative Oncology Group (ECOG) Performance Status (PS) of 3–4 had a small CAR (p<0.001) and flatter diurnal cortisol slope (p<0.001) compared to than patients with an ECOG PS of 1–2. Patients with metastatic disease had a small CARauc than those without metastatic disease (p=0.003). Total MD Anderson Symptom Inventory scores, fatigue, and interference with general activity, work, and walking were all significant associated with a reduced CAR and a flatter diurnal cortisol slope (p<0.05).
Rebholz et al., 2018 (65)	57 (F) stage 0–5 breast cancer patients, mean age 52.32	Combination: 3-day salivary cortisol (waking, +30 min, 16:00, bedtime) and 3-day actigraphy (AMI)	The diurnal cortisol slope was not significantly correlated with QoL.
Cash et al., 2015 (15)	43 (F), stage 0–4 breast cancer patients, mean age 52.49	Combination: 3-day salivary cortisol (waking, +30 min, 16:00, “prior to going to bed”) and 3-day actigraphy (Micro Mini-Motionlogger) and sleep diary	A higher CAR was associated with elevated levels of VEGF, TGF-beta, and MMP-9 (markers associated with angiogenesis, immunosuppression, epithelial–mesenchymal transition, tumour invasion and metastasis).
Hsiao et al., 2015 (66)	62 (F) stage 0–3 breast cancer patients, 25.8% had metastatic disease, mean age 35.3	24-h salivary cortisol (waking, +30 min, +45 min, 12:00, 17:00, 21:00)	A flatter diurnal cortisol slope was associated with greater tumour size (p=0.01) an increase of body mass index over 8 months (p=<0.001) and a persistently later waking time over 8 months (p=0.006). Factors that were not significantly associated with the cortisol slope included metastatic status, physical activity levels, time of going to bed, sleep problem index, and depressive symptoms.
Schrepf et al., 2015 (18)	113 (F) stage 1-4 ovarian, primary peritoneal or fallopian tube cancer patients, mean age 57.99	Salivary cortisol (waking, 17:00, and bedtime)	Increasing age was associated with a higher evening cortisol (p=0.004) but not cortisol variability or slope. “High grade” disease, and poorer physical well-being were associated with a higher night cortisol, a flattened diurnal cortisol slope, and reduced cortisol variability (all p<0.05). “Late” stage disease was also associated with higher evening cortisol (p=0.05). Shorter survival was seen with elevated night cortisol prior to surgery (HR, 1.802, p<0.001), and the diurnal cortisol slope (HR, 1.633, p=0.001). Longer survival was seen with cortisol variability (HR, 0.644, p<0.001). Estimated median survival for low evening cortisol was 7.3 years compared to 3.3 years in those with a high evening cortisol. Elevated night cortisol, a flattened diurnal cortisol slope, and reduced cortisol variability were associated with higher levels of inflammation indicated by ascitic and plasma IL-6 (all <0.05). The diurnal cortisol slope was associated to cortisol variability (r = 0.88, p<0.001). Night cortisol was correlated with cortisol variability (r=−0.727, p<0.001) and the diurnal cortisol slope (r=0.758, p<0.001).
Zeitzer et al., 2015 (16)	97 (F) recurrent or metastatic breast cancer patient, “age” 57.4	Combination: 27-h inpatient serum cortisol (20–60-minintervals from 3 h prior to bedtime to 1 h after wake time), salivary cortisol 09:00 on day 1, awakening and 30 min on day 2 and 2-week outpatient actigraphy (actiwatch 2) and sleep diary logs	The cortisol diurnal slope varied depending on the analytical method used. 10/91 (11%) had a “positive” slope from 06:00. A flatter diurnal cortisol slope was associated with a lower morning peak and elevated evening trough. Plasma and salivary cortisol concentrations were correlated (p<0.001).
Diaz et al., 2014 (67)	99 (F) metastatic breast cancer patients, median age 54	2-day salivary cortisol (waking, +30 min, 12:00, 17:00, and 21:00)	Post-traumatic growth (positive psychological change following cancer diagnosis or treatment) was associated with a steeper cortisol slope (p=0.039). ‘Relating to others’ subscale was also associated with a steeper cortisol slope (p=0.039). Income, education, marital status, age, time since recurrence, PR status, and metastatic sites were unrelated to cortisol slope (p>0.05).
Palesh et al., 2014 (68)	97 (F) metastatic or locally advanced breast cancer patients, mean age 54.6	Combination: 2-day salivary cortisol (waking, +30 min, 12:00, 17:00, and 21:00) 3-day actigraphy (micro-mini-motion logger) and sleep diary	Salivary diurnal cortisol was associated with survival (HR, 1.03; p=0.85) (character of the slope was not noted).
Sephton et al., 2013 (69)	62 (34 F) stage 1–4 non-small cell lung cancer and small cell lung cancer patients, mean age 64	2-day salivary cortisol (waking, +45 min, 16:00, 21:00) Single serum cortisol (time not defined)	A lack of normal diurnal variation was associated with shortened survival (HR, 68,052.8; p=0.009). The cortisol AUC and CAR were not associated with survival time. Flattened cortisol rhythms were associated with more advanced lung cancer (r=0.35, p=0.003), poor performance status (r=−0.29, p=0.012), being male (p=0.028), low total lymphocytes (r=−0.39, p=0.002), and low cytotoxic T lymphocyte count (r=−0.30, p=0.017). The diurnal cortisol slope was not significantly associated with CAR, age at diagnosis, cancer type, time since radio- or chemotherapy, socioeconomic status, prior martial disruption, depressive symptoms, fatigue, or sleep difficulties. The diurnal salivary cortisol slope was not associated with serum cortisol levels.
Cohen et al., 2012 (70)	202 (156 M), metastatic renal cell cancer patients, mean age 59	3-day salivary cortisol at waking +45 min, 8 h, 12 h, and bedtime)	The cortisol slope was significantly associated with survival (HR, 1.88; p=0.002). The cortisol slope was not associated with psychosocial variables or CES-D scores.
Dedert et al., 2012 (71)	57 (F) stage 1–4 breast cancer patients, mean age 52	Combination: 3-day salivary cortisol (3 days—waking, 30 min after, 16:00 and bedtime) and actigraphy and sleep diary	Intrusion and avoidant coping were not related to cortisol measures. A higher autocorrelation coefficient (a measure of physical activity circadian consistency between days) was associated with a steeper diurnal cortisol slope (r=−0.41, p=0.003).
Brivio et al., 2010 (72)	14 (10 M) metastatic non-small cell lung cancer, pancreatic cancer, prostate cancer, and malignant melanoma patients, median age 67	Serum cortisol (08:00 and 16:00) at baseline and following melatonin treatment	Patients had abnormal cortisol circadian rhythms (defined as a lack of decline of 30% in the cortisol level from the morning to the afternoon). No significant difference was reported in morning or afternoon mean cortisol levels between stable and progressive disease (significance levels not reported).
Sephton et al., 2009 (73)	72 (F), stage 4 metastatic breast cancer, mean age 54.5	3-day salivary cortisol (08:00, 12:00, 17:00, 21:00)	Greater depression symptoms were associated with higher morning cortisol (p<0.02) and accentuated diurnal cortisol rhythms (p≤0.05). Depression scores were uncorrelated with mean cortisol levels.
Lutgendorf et al., 2008 (74)	25 (F) low malignant potential patients, mean age 51.24 26 (F) early-stage ovarian cancer patients, mean age 55.6 86 (F) advanced ovarian cancer patients, mean age 60.22	Salivary cortisol (waking, +30 min, 15:00–18:00, 20:00–24:00)	Salivary cortisol was increased for all patients with advanced cancer patients having approximately 3 times the healthy population normal values. Cortisol AUC was significantly higher in advanced-stage patients compared to the low malignant potential patients (p=0.047). Diurnal cortisol levels did not significantly differ between groups or over the day (p>0.06 and p>0.73). Higher evening cortisol levels were associated with higher total depression (p=0.026) and vegetative depression (p=0.005).
Palesh et al., 2008 (75)	99 (F), metastatic breast cancer patients, mean age 54.65	Combination: 2-day salivary cortisol (waking, +30 min, 12:00, 17:00, 21:00) and 3-day actigraphy (micro-mini-motionlogger)	A flatter cortisol slope was associated with longer average wake episodes at night (r=0.21, p=0.04). No significant relationship was found between mean waking cortisol or cortisol rise and other sleep measures.
Giese-Davis et al., 2006 (14)	29 (F) stage 4 breast cancer patients, mean age 53.21 All received supportive-expressive group therapy (SET)	3-day salivary cortisol (08:00, 12:00, 17:00, 21:00)	Steeper diurnal cortisol slopes were associated with shorter duration of negative affect (p=0.02). 08:00 and mean cortisol levels were not associated.
Jehn et al., 2006 (19)	114 (38 M), mixed stage 4 cancer patients, (71 had “progressive” disease, 43 had “stable” disease) Mean age of patients with depression 62.7 Mean age of patients without depression 59.4	Serum cortisol (08:00 and 20:00)	Cortisol concentrations were significantly higher in patient with depression at 08:00 (p=0.003) and 20:00 (p<0.001). Cortisol diurnal variation (VAR) was significantly decreased in cancer patients with depression compared to those without depression (11.7% vs. 60.6%, p = 0.001).
Mussi et al., 2006 (76)	40 (22 F), colorectal cancer patients (13 patient had nodal disease, 10 had liver metastases), median age 66	Serum cortisol (23:00 and 08:00)	Patients with liver involvement had a higher evening cortisol (p<0.0005). Nodal involvement did not impact on cortisol levels. 28% had an altered circadian rhythm defined as 23:00 level >50% of 08:00 level. This was more frequent if there was nodal involvement and metastatic spread (p<0.005). Cortisol levels and circadian rhythm were unrelated to CD4+ lymphocyte count (a prognostic marker).
Spiegel et al., 2006 (77)	99 (F) metastatic breast cancer patients, median age 54	2-day salivary cortisol (waking, +30, 12:00, 17:00, 21:00)	The cortisol slope was correlated with cortisol rise within 30 min of waking (r=0.29, p=0.004), but not with the waking level (p=0.19). A flatter slope was associated with a higher 21:00 level (r=0.85, p<0.0001) and escape from cortisol suppression (r=0.30, p=0.005). No significant association was reported between the cortisol slope and CRF administration or social stress. Antidepressant use was associated with higher waking cortisol (r=0.21, p=0.04) and lower cortisol rise (r=−0.32, p=0.001). Lower income status was associated with flatter cortisol slope (r=−0.28, p=0.008). Patients with progesterone receptor positive breast cancer had a lower waking cortisol rise (r=0.22, p=0.04). The cortisol slope was unrelated to demographics, disease-free interval, or treatment. Patients with progesterone receptor positivity had lower waking cortisol rise (p=0.04).
Rich et al., 2005 (78)	80 (52 M) metastatic colorectal cancer patients Group 1—40 patients with a “good” rhythm (r24 >0.47 <0.77), median age 59.6 Group 2 - 40 patients with a “dampened” rhythm (r24 >0.03, <0.35), median age 60	Combination: 2-day serum cortisol (08:00 and 16:00) and 3-day actigraphy (Actigraph)	Patients with a “good” activity rhythm had higher cortisol ratios (between 08:00 and 16:00) compared to those with a “dampened” activity rhythm. Mean cortisol levels did not differ significantly between groups. Mean cortisol was prognostic (p<0.0001). IL-6 and TGF-alpha were positively correlated with mean serum cortisol (p=0.0001). IL-6 was negatively correlated with circadian cortisol ratio (p = 0.042).
Giese-Davis et al., 2004 (79)	91 (F) metastatic breast cancer patients, mean age at diagnosis of metastatic disease 49.08-55.93	3-day salivary cortisol (mean times 08:06, 12:26, 17:25, 20:26)	40% patients displayed the usual morning cortisol peak with a decline across the day. 56% had peaks later in the day and 4% had flattened rhythms. Patient’s demonstrating psychological repression had flatter slopes than the self-assured (p=0.01) and non-extreme groups (p=0.02). Mean cortisol levels were not significantly different between groups.
Mormont et al., 2002 (80)	Round the clock study (RTCS): 18 (14 M) metastatic colorectal cancer patients, mean age 58 Two-time point study (TTPS): 192 (128 M) metastatic colorectal cancer patients, mean age 58	RTCS: Cortisol (13 blood and salivary samples 1st and 4th day of 4-day chemotherapy) TTPS: Cortisol (2 day—blood and saliva 08:00 and 16:00, + saliva 23:00 all before chemotherapy)	A significant circadian rhythm was seen in the serum of 8/18 (44.4%) patients and in the saliva of 6/16 patients (37.5%). Patients with marked circadian rhythms had lower 08:00 and 16:00 levels. Marked cortisol rhythms were not associated with longer survival than those with altered rhythms and cortisol did not predict the clinical outcome. A higher performance status and per cent of liver replacement was associated with higher cortisol concentrations at 08:00 and 16:00. Salivary and serum cortisol were correlated, particularly for those with stronger circadian rhythms.
Mormont et al., 2002 (49)	18 (14 M) metastatic colorectal cancer patients, mean age 58	Combination: salivary cortisol and serum cortisol, melatonin and 6-alphasulfatoxymelatonin (3–6 hourly over 10–13 time points on day 1 and 4) and 3-day actigraphy (Actigraph)	A circadian cortisol rhythm was seen in serum cortisol for 8 patients, and 6 patients had a significant salivary cortisol rhythm. Interindividual variation in markers of circadian rhythm.
Mormont et al., 2000 (37)	192 (128 M) metastatic colorectal cancer patients, mean age 58	Combination: 2-day blood cortisol (08:00 and 16:00) and 3-day actigraphy (actigraphy) and sleep diary	Mean cortisol was higher in patients with a low r24 (a measure of physical activity circadian consistency) (r=−0.17, p=0.04), low I<O (a measure of activity in and out of bed) (r=−0.24, p=0.07), poor performance status (p=0.0005) or severe liver involvement (p=0.0001). The estimate of cortisol circadian rhythm (difference in cortisol values) was correlated with r24 (r=0.16, p=0.04) but not I<O or mean activity. Cortisol was not prognostic, and the circadian rhythm did not estimate treatment response.
Sephton et al., 2000 (81)	104 (F), metastatic breast cancer patients, mean age 53.2	3-day salivary cortisol (08:00, 12:00, 17:00, 21:00)	Flatter diurnal cortisol slopes were associated with shorter survival (HR, 464.9; p = 0.0036). The change in survival between slopes was seen up to 7 years later. The diurnal cortisol slopes were split at the median value, 77% with “flat” rhythms averaged 3.2 years survival, whereas 60% with “steep” rhythms averaged 4.5 years survival. The diurnal cortisol slope remained prognostic after adjusting for age at initial diagnosis, disease-free interval, and oestrogen receptor status. Flatter diurnal cortisol slopes were associated with taking megestrol (p=0.000), more nocturnal awakenings (p=0.003), marital disruption (p=0.040), fewer circulating NK cells (p=0.007), and suppressed NK cell activity (p=0.05). Steeper slopes were associated with metastases to the chest wall or adjacent lymph nodes (p=0.023).
Mormont et al., 1998 (50)	18 (14 M) metastatic colorectal cancer patients, age 35–72	Combination: 6 hourly blood for cortisol and melatonin (9–11 time points) on day 1 and 4 and 3-day actigraphy	Study 1: 7 of 18 (39%) patients had significant cortisol circadian rhythms (p<0.05). Group circadian rhythms for cortisol were validated (p<0.00001). Wide interindividual variation was noted between cancer patients.
Touitou et al., 1996 (36)	13 metastatic breast cancer patients, mean age 52 (gender not defined) 20 stage 2a–4 ovarian cancer patients, mean age 57	24-h serum cortisol (4 hourly intervals)	Serum 08:00 cortisol was low in 9 patients. 8 breast cancer patients had flattened cortisol patterns, a shift in the peak or trough time, or a plateau with high morning values. 15 ovarian cancer patients had high cortisol levels throughout a 24-h period and/or erratic peak and trough locations and/or flattened profiles.
Touitou et al., 1995 (82)	13 (F) stage 1–4 metastatic breast cancer, median age 52	48-h serum cortisol (4 hourly intervals)	The cortisol acrophase was near 0930 ± 110. A significant circadian rhythm was seen in patients with WHO PS1 or no liver metastases. Circadian cortisol rhythm was lost for patients with liver metastases.
Touitou et al., 1990 (83)	13 (F) metastatic breast cancer patients, mean age 52	36–48-h serum cortisol (4 hourly intervals)	6 patients had normal rhythmicity with a peak at 08:00 and trough 00:00. 7 patients had altered peak and/or high concentration between 04:00–12:00 and/or flattened rhythms.

**Table 2C:** Actigraphy-related circadian rhythms and their associations in patients with advanced cancer.

Studies with a control group			
Article	Participants	Circadian measures	Outcomes relevant to review
Levi et al., 2020 (17)	Combined data IDEAs study: 25 (21 M) locally advanced or metastatic gastrointestinal cancer patients, median age 66 PicaPill study: 33 (15 M), control subjects, median age 35	7-day actigraphy and chest accelerometry and 2-day salivary cortisol (3-hourly) and 1-day melatonin samples (hourly from 19:00)	13 patients had a dichotomy index (I<O, ratio of activity in and out of bed) of ≤97.5% on chest accelerometry. Patients had worse I<O (p=0.008), levels of activity (p<0.0001), and rest probability P1-1 (probability of remaining in a rest state) than controls (p=0.005). The activity amplitude (between peak and trough activity levels), r24 (autocorrelation coefficient, a measure of physical activity consistency between days), RI (rhythm index, measure of quality, regularity, and consistency of rest state), average centre-of-rest time, and rest duration were not significantly different. Hospital Anxiety and Depression Scale (HADS) scores, performance status, and Pittsburgh Sleep Quality Index (PSQI) scores did not differ significantly between I<O groups. Large inter-subject variability was noted. 91% of patients with a I<O >97.5% were physical active 30 min/+ a day compared to 15% of patients with a I<O of ≤97.5% (p=0.001). Controls aged 40/+ had a reduced I<O (p=0.01), P1-1 (p=0.0009) and phase-advanced activity acrophase (earlier peak activity time) and centre-of-rest time (p=0.01). Patients aged 40/+ had similar I<O values to older controls. For patients, the I<O was associated with day-to-day variability in sleep duration (r=−0.53, p=0.009), self-reported exercise (r=0.48, p=0.02), rest–activity amplitude (r=0.73, p<0.0001), median activity out-of-bed (r=0.68, p=0.0003), level of activity (r=0.56, p=0.005), day-to-day variability in self-reported retiring time (r=0.49, p=0.02), physiological chest temp (p=0.03), and chronotype (a measure of sleep timing preference) score (r=−0.43, p=0.04). The circadian amplitude in rest–activity and sleep duration variability were the best predictors of a patient’s I<O.
Zeitzer et al., 2016 (52)	97(F) recurrent or metastatic breast cancer patients, age 57.6 24 health age-matched controls, age 57.1 (gender not defined)	2-week actigraphy (Actiwatch 2) and sleep diary & 28-h plasma cortisol (20–60-min intervals) and polysomnography	No reported associated with actigraphy derived data.
Natale et al., 2015 (28)	226 (149 M) metastatic colorectal cancer patients, mean age 58.4 182 (103 F) control subjects, mean age 38.7	At least 4-day actigraphy (Basic Mini-Motionlogger)	Patients had a lower median I<O than controls (97.8% vs. 99.6%) The lowest patient I<O was 75.7% compared to 97.2% in controls. Patients spent a longer time in bed (67 min longer on average) than controls but slept a similar amount of time. Patients had longer sleep onset latency, mean sleep motor activity, wake after sleep onset, number of awakenings more than 5 and lower sleep efficiency compared than controls. I<O was significantly correlated with sleep motor activity (movements during sleep within a given time frame), wake after sleep onset, number of awakenings more than 5 and sleep efficiency (all p=0.0001). Younger patients went to bed, and woke up, later than older patients, and had a delay in the midpoint of sleep. The actigraphic parameter to best discriminate cancer patients was I<O.
Grutsch et al., 2011 (30)	84 (65 M), stage 2b–4 non-small cell lung cancer patients, mean age 62 35 control subjects from the Ambulatory Monitoring Inc. database (not further defined)	2-7-day actigraphy (Mini Motionlogger Basic Model 4)	Actigraphy parameters were abnormal for all cancer patients compared to controls. Same figures for actigraphy parameters as Du-Quiton et al., 2010). Higher daytime activity was associated with lower PSQI daytime dysfunction (r=−0.61, p=0.006), higher PSQI sleep quality (r=−0.48, p=0.014), and less use of self-reported sleep medication (r=−0.58, p<0.003). Higher daytime inactivity was associated with more daytime dysfunction (r= 0.54, p=0.017), lower PSQI global sleep quality (r=0.41, p=0.014), and higher self-reported use of sleep medication (r=0.39, p=0.05). A higher 24-h rhythm quotient was associated with less daytime dysfunction (r=−0.58,2p <0.01). Patients who slept well during the night and less in the day slept for longer regardless (p<0.03) Higher levels of night-day sleep balance were associated with less nighttime sleep disturbance (r=−0.44, p=0.067), less daytime dysfunction (r=−0.43, p=0.065) and better global PSQI sleep (r=−0.36, p=0.071)
Grutsch et al., 2011 (20)	84 (65 M) stage 2b-4 non-small cell lung cancer patients 42 inpatients, mean age 57 42 outpatients, mean age 66 35 control subjects from reference database, age 20-50 (gender not defined)	2–7-day actigraphy (Ambulatory Monitoring Inc.)	All actigraphy parameters were abnormal in patients. A flatter activity circadian rhythm was observed for patients. A higher r24 was associated with less insomnia (r=−0.48, p=0.003) Loss of appetite was associated with decreased peak activity (r=−0.41, p=0.005) and the circadian quotient (the amplitude/MESOR) (r=0.4, p=0.015) A higher r24 was associated with higher quality-of-life index Health/Functioning domain scores (r=0.34, p=0.05). Higher satisfaction with health was associated with more stable circadian structures. Fatigue was associated with a diminished circadian quotient (r=−0.40, p=0.04), rhythm quotient (4=−0.41, p=0.03) and night-day sleep balance (r=−0.52, p<0.01). A more robust rhythm was associated with less fatigue. A higher rhythm quotient was associated with less pain (r=−0.39, p=0.04). Loss of appetite was negatively associated with night-day sleep balance (r=−0.47, p<0.01). Peak activity significantly correlated with all the Power and Ferrans QoL index domains (all p≤0.02). Robustness of circadian measures reflects all quality-of-life measured aspects. Higher r24 was associated with higher health/functioning domain scores (r=0.45, p=0.02) and global health scores (r=0.53, p<0.01). Night-day sleep balance was correlated with the health/functioning domain (r=0.39, p=0.04), social/economic domain (r=0.40, p=0.04) and psychological/spiritual domain (r=0.45, p=0.02). A larger difference in nocturnal and daytime activity correlated with the score in each QLI measure. Night-day balance was correlated with the EORTC QLQ C30 domains of role (r=0.56, p<0.01) and cognitive function (r=0.45, p=0.02). A more robust circadian rhythm was associated with greater patient satisfaction with health/functioning, and better overall quality of life.
Du-Quiton et al., 2010 (23)	84 (65 M) stage 2b–4 non-small cell lung cancer patients 42 inpatients, mean age 57 42 outpatients, mean age 66 Control subjects from the Ambulatory Monitoring Inc. database	3–7-day actigraphy (Ambulatory Monitoring Inc. action W-2)	All actigraphy parameters were abnormal for cancer patients compared to controls (p<0.05). Patients were 20%–50% less active than controls. Patients had at least 3 times longer daytime inactivity, 4 times longer sleep/inactivity periods in the day (20.9% vs. 4.7%) than controls. Patients had more fragmented sleep, longer waking episodes and less nighttime sleep than controls. The longest patient night sleep episode was less than half of the controls. Patients had lower sleep efficiency (79.8% vs. 95.9%), shorter longest nighttime sleep duration (91.7 min vs. 255.6 min), less activity (126.9 accelerations/min vs. 182.6), shorter daytime wake time (797.5 min vs. 947.1 min), shorter sleep time at night (284.0 min vs. 417.8 min), and shorter % of time asleep at night (72.5% vs. 93.0%) than controls (all p<0.05). Patients took longer to fall asleep at night (20.8 min vs. 12.1 min), were awake more in the night (95.0 min vs. 31.1 min), slept for longer in the day (208.8 min vs. 47.1 min), and had longer longest daytime sleep periods (43.0 min vs. 23.6 min) than controls (p<0.05). Overall daytime and nighttime sleep were not associated with anxiety or depression. There were no statistically significant associations between sleep-activity circadian rhythm and anxiety or depression amongst inpatients.
Fouladiun et al., 2007 (84)	39 (28 M) mixed cancer patients (32 metastatic), mean age 71 31 control subjects, two cohorts mean ages 49 and 74 (genders not defined)	≥3-day actigraphy (Actigraph)	Patients were less active in the day on weekdays and weekends than controls (p<0.01). Physical-rest activity was not different to age-matched and recently hospitalised non-cancer patients. Subjectively scored physical function and pain were predicted by objectively measured physical activity (p<0.0001).
Le Guen et al., 2007 (85)	29 (25 M) stage 1–4 limited and extensive small cell lung cancer patients, mean age 59 14 (12 M) obstructive sleep apnoea patients treated with nasal continuous positive airway pressure, mean age 55	5-day actigraphy (Actiwatch) and sleep log	Actigraphy parameters differed between the two groups. During the night, cancer patients had longer sleep times (7.4h vs. 6.5h, p=0.03), lower sleep efficiency (78% vs. 88.1%, p=0.002), higher mean activity scores (31.5 vs. 9.6, p<0.001), a higher fragmentation index (not described) (51.7 vs. 28.4, p=0.002), and lower immobile time (75.2% vs. 87.4%, p=0.004). Sleep latency was not significantly different between groups. Patients had lower daytime mean activity scores than controls (186.5 vs. 274.8, p=0.001) The PSQI and ESS scores were not significantly correlated with any actigraphic parameters.
Pati et al., 2007 (86)	31 (19 M) stage 2–4 mixed cancer patients, median age 43 35 (22 M) control subjects, median age 35	4-day actigraphy (Actiwatch) and sleep diary	Activity levels were higher, activity patterns more regular, and day–night activity more distinct in controls compared to patients. Cancer patients demonstrated more frequent episodes of activity. Activity rhythms occurred in a 24-h pattern for most participants except 4 patients and 1 control who had activity rhythms lasting 6 or 12 h. MESOR, amplitude and I<O were lower in patients than controls (all p<0.001). The activity acrophase occurred early (~1 h) in patients than controls (p<0.001). The mean r24 of the patient group was lower, but not significantly different, than the control group. Patients spent more time in bed (p<0.02), had more wake episodes (p<0.001), a higher nap frequency (p<0.001), more total naps (p<0.001), and longer naps (p<0.001) than controls.
Fernandes et al., 2006 (32)	25 (F) mixed cancer diagnoses patients (72% metastatic) median age 67 25 (F) control subjects, median age 63	3-day actigraphy (Actimeters) and sleep diary	All actigraphy parameters were significantly different between the two groups. Patients had lower median r24 values (0.28 vs. 0.46, p<0.001), longer wake after sleep onset (68.34 min vs. 25.67 min, p=0.004), and lower sleep efficiency (85.75% vs. 94.63%, p=0.001). Sleep latency was not significantly different between groups. Actigraphy parameters were not significantly correlated with EORTC QLQ-C30 fatigue score in patients or controls.
Levin et al., 2005 (87)	33 stage 3–4 non-small cell lung cancer patients, 2 cohorts MRMC site: 21 (12 F), mean age 56.71 VAMC site: 12 (M) mean age 71.75 35 control subjects	4–7-day actigraphy (Ambulatory Monitoring Inc.)	Actigraphy parameters were significantly different between patients and controls. The I<O for the patient cohorts was 90.0±2.2 and 78.9 ± 2.4. During wakeful periods patients had less daily activity (mean 92.8 min vs. 127 min), more sleep time (mean 195 min vs. 46.5 min and % asleep 21.8 vs. 4.7), and more sleep episodes (17.8 vs. 5.4) than controls. During nighttime periods, cancer patients spent more time awake (134.1 min vs. 31.1 min), had more sleep interruptions (14.6 vs. 6.9), and spent less time sleeping (71.2% vs. 93.0%). The r24 varied depending on the ECOG PS (PS0 0.31, PS1 0.17, PS2 0.21) The duration of long sleep episodes was longer in PS0 patients than PS2 (129 min vs. 96.5 min, p<0.05). No significant differences in I<O, peak activity and the circadian quotient were seen between groups. The ultradian (4 hourly rhythms) were significantly different (p=0.046). Circadian quotient was lower with a higher PS (PS0 0.55±0.08, PS1 0.53±0.04, and PS2 0.47±0.06). Rhythm quotient was lower with a higher PS (PS0 1.05±0.16, PS1 1.02±0.09, and PS2 0.94±0.16).
Chevalier et al., 2003 (88)	10 (5 M) metastatic colorectal cancer patients, median age 61 15 (M) control subjects (age not defined)	3-day actigraphy (Actigraph)	The median r24 for controls was 0.57 and 1 control had an r24 <0.40. Mean activity counts ranged from 118 to 163 with a median of 145. The peak activity time (acrophase) ranged between 14:20 and 20:10 with a median acrophase of 17:00. The median r24 for patients was 0.57 and 2 patients had an r24 <0.40. Mean activity counts ranged from 76 to 275 counts with a median 148. The acrophase ranged from 13:18 to 17:54 with a median acrophase of 15:24.
Studies without a control group			
Patel et al., 2023 (89)	44 (17 F) mixed locally advanced or metastatic cancer diagnoses, median age 66	72-h actigraphy (Actiwatch)	Mean I<O 88.9% (70.9%–98.1%) I<O correlated with r24, mean activity, sleep efficiency, WASO I<O not associated with survival (based on median or quartile groups) I<O negatively correlated with ECOG-PS (p<0.0005) Autocorrelation coefficient 0.16 (0.04–0.37), unrelated to survival No actigraphy-derived sleep parameters associated with survival Sleep efficiency and later get up time associated with survival
Block et al., 2022 (90)	30 (22 F), stage 2–4 mixed cancer diagnosis, median age 55	7-day actigraphy (Ambulatory Monitoring Inc) Day 6 overnight and morning urine 6-sulfatoxymelatonin	Baseline actigraphy from 22 patients r24 median 0.48, mean 0.47 (0.08–0.63) Intradaily stability median 0.73, mean 0.73 (0.43–0.86) Intradaily variability median 0.48, mean 0.53 (0.37–0.88) Relative amplitude median 0.87, mean 0.86 (0.76–0.95)
Padron et al., 2022 (91)	Cognitive behavioural therapy for insomnia and pain (CBTi.p).: 18 (F) stage 1–3 mixed gynaecological cancer patients, mean age 58.9 Psychoeducation (PE): 17 (F) stage 1–4 mixed gynaecological cancer patients, mean age 59.9	14-day actigraphy (Actiwatch-L) and sleep diary a post-surgical polysomnography	Actigraphy and sleep diary data were not correlated. Baseline mean values (polysomnography vs. actigraphy) CBTi.p.: sleep efficiency was 75.5% vs. 80.8%, sleep onset latency (time between being in bed and falling asleep) was 24.7 min vs. 29.3 min, and wake after sleep onset was 91.1 min vs. 50.5 min PE: sleep efficiency was 71.0% vs. 79.4%, sleep onset latency was 36.2 min vs. 33.6 min, wake after sleep onset was 94.8 min vs. 53.4 min Baseline objective data (CBT.i.p and PE): 29% and 24% of patients had a sleep efficiency >85% 76% and 50% of patients had sleep onset latency <30 min 6% and 13% of patients had wake after sleep onset <30 min
Studies without a control group			
Article	Participants	Circadian measures	Outcomes relevant to review
Cheung et al., 2021 (10)	30 (16 M) stage 3b–4 non-small cell lung cancer patients Group 1—Aerobic exercise, mean age 61.00 Group 2—Tai-Chi, mean age 61.11 Group 3—Self-management group, mean age 58.36	3-day actigraphy (Ambulatory Monitoring Inc.) and salivary cortisol rhythms (0.5, 4, 8, and 12 h after waking)	Patients had a total sleep time of (1) 283.03 min, (2) 240.46 min, and (3) 295.48 min. Sleep efficiency (total sleep time to time in bed ratio) was (1) 91.13%, (2) 89.69%, and (3) 90.70%
Bernatchez et al., 2020 (92)	57 (30 M) mixed cancers patients (45 stage 3–4, 53 metastatic), mean age 65.8	7-day actigraphy (Actiwatch-64) and sleep diary	No objective measures were significantly correlated with time to death or quality of life (QoL). Higher sleep efficiency was associated with less pain and depressive symptoms (all r=−0.23, p≤0.05). Longer napping time was associated with increased pain (r=0.38, p≤0.01), fatigue (r=0.37, p≤0.01), daytime sleepiness (r=0.38, p≤0.01), and depressive symptoms (r=0.23, p≤0.05). Gastrointestinal symptoms were associated with increased sleep onset latency (r=0.33, p≤0.01), nighttime awakenings (r=0.30, p≤0.05), and time in bed (r=0.34, p≤0.01). Maladaptive sleep behaviours were associated with longer sleep onset latency (r=0.27, p≤0.05), increase nighttime awakening (r=0.24, p≤0.05), early morning awakening (r=0.24, p≤0.05), and poorer sleep efficiency (r=−0.35, p≤0.01). None of the objective measures were significantly correlated to erroneous sleep beliefs or 24-h light exposure.
Jakobsen et al., 2020 (29)	40 (24 M), metastatic mixed cancer patients, median age 70	Overnight actigraphy (Actiwatch 2) and polysomnography	Actigraphy: mean total sleep time was 418 min (SD±138), mean sleep onset latency was 35 min (SD±61), mean number of awakenings was 24 (SD±15), mean total time awake was 40 min (SD±21), and mean per cent of time in bed asleep was 78% (SD±23). Actigraphy measured total sleep time (r_s_=0.61, p<0.005) and sleep efficiency (r_s_=0.48, p<0.005) were associated with patient reported outcomes measures (PROMs). Sleep onset latency, number of awakenings, and wake after sleep onset were not associated with PROMs. Longer wake after sleep onset (actigraphy) was “significantly” associated with worsening of total subjective sleep quality (r_s_ = 0.45, significance level not reported).
Fujisawa et al., 2019 (31)	51 (20 M), stage 4 non-small cell lung cancer patients, mean age 66.8	3-day actigraphy (Actiwatch 2) and activity log	The hazard ratio was 1.48 for each 10% increment of time awake spent immobile (p<0.05). The odds ratio for death within 6 months was 2.99 for each 10% increment of time awake spent immobile (p<0.05). Eastern Cooperative Oncology Group (ECOG) performance status (PS) was non-discriminatory for survival in patients with a PS 0–1. For those who died at 6 months, discriminating factors for survival were 10% increment of time awake spent immobile (OR 2.99, p<0.05), ECOG PS >1 (OR 9.23, p<0.05), and the percentage of time awake spent immobile (OR 5.09, p<0.05). Time awake spent immobile correlated with Functional Assessment of Cancer Therapy—Trial Outcome Index (FACT-TOI) (p<0.01) and ECOG PS (p<0.01).
Komarzynski et al., 2019 (27)	31 (17 M), mixed cancer patients (24 metastatic), median age 61	30-day actigraphy (Micro Motionlogger)	Median sleep efficiency was 92.0% (20.2%–100%), the sleep midpoint was 03:29 (00:38–10:19), wake time was 08:00 (03:49–15:39), bedtime was 23:07 (19:29–05:00), sleep onset latency was 5 min, wake after sleep onset was 42 min, number of wake episodes was 11, and total sleep time was 7hr50 (6h50–8h44). Subjective sleep disturbance was associated with objective parameters (p≤0.05). Worse MD Anderson Symptom Inventory (MDASI) sleep scores were found in those with lower sleep efficiency (r=−0.13, p=0.002), larger number of wake episodes (r=0.12, p=0.005), longer wake after sleep onset (r=0.14, p<001), and worse sleep fragmentation index (r not defined) (p=0.01). The number of wake episodes was associated with fatigue (p=0.02), drowsiness (p=0.03) and interference with activity (p=0.03). Sleep efficiency was associated with daytime drowsiness (p = 0.01). MDASI sleep correlated with sleep efficiency, wake minutes, wake episodes, sleep episodes, and longest sleep episodes (all p<0.01). MDASI sleep correlated with wake after sleep onset (p<0.001). MDASI fatigue correlated with total sleep time, total time in bed and sleep episodes ≥5 min (all <0.01). MDASI drowsiness correlated with wake after sleep onset and total time in bed (all p<0.01). MDASI interference with activity correlated with sleep episodes ≥5 min and longest sleep episode (all p<0.01).
Bernatchez et al., 2018 (8)	55 (29 M) mixed cancer patients (44 stage 3–4, 52 distant metastases), mean age 65.9 Actigraphy data available for 55 patients, outliers removed and 51 analysed	7-day actigraphy (Actiwatch-64) and sleep diary	The R-squared (rhythmicity coefficient of the sleep–wake cycle) was 0.27 (0.09–0.51), acrophase was 13:35 (10:34–20:10), MESOR (average activity level) was 45.4 (3.6–167.8), amplitude was 47.0 (5.4–178.8), up-MESOR (mean time from low to high activity) was 8.18 (2.00–14.00), and down-MESOR (mean time from high to low activity) was 19.23 (16.20–22.00). Less rhythmic sleep–wake cycle, as characterised by lower amplitude (r=0.32), MESOR (r=0.27), and R-squared (r=0.31), was associated with shorter survival (all p≤0.05). No circadian activity rhythm parameter was correlated with pain, fatigue, depression, or maladaptive sleep behaviours. A lower amplitude (r=0.24, p≤0.01), lower MESOR (r=0.27, p≤0.05), and later acrophase (r=−0.23, p≤0.01) was associated with poorer global QoL. A higher down-MESOR was associated with poor global (r=−0.31, p≤0.05) and functioning QoL (r=−0.30 p≤0.05). A more robust rest-activity rhythm (higher amplitude (r=0.33, p≤0.05), MESOR (r=0.42, p≤0.01), and r-squared (r=0.24, p≤0.05) was associated with greater 24-h exposure to light intensity >1,000 lux. There were no significant differences between any rest–activity rhythm variable between ECOG 2 vs. 3.
Bernatchez et al., 2018 (25)	57 (26 M) mixed cancer patients (40 stage 3–4, 48 distant mets) 51 analysed, mean age 66.4	7-day actigraphy (Actiwatch-64) and sleep diary	The wake after sleep onset ranged between 48.2 and 70.9 min. The longest daytime napping was 100.3 min. Patients with no perceived sleeping difficulty had a mean sleep onset latency of 10.2 min, wake after sleep onset of 48.2 min, total wake time of 68.5 min, total sleep time of 479.9 min, time in bed of 548.4 min and sleep efficiency of 87.3%. Subjective assessments differed from objective assessment, significance not reported. No significant differences for sleep-wake parameters were noted between men and women.
Cash et al., 2018 (93)	55 (33 M) stage 0–4 head and neck cancer patients, mean age at diagnosis 58.5	6-day actigraphy (Mini-Motion logger) and sleep diary	The mean rest–activity rhythm (RAR) correlation coefficient (r24) was 0.132 and mean nighttime restfulness was 92.82%. The activity acrophase occurred at 14:46. 2-year survival was impacted by RAR disruption (HR, 0.073; p = 0.012), lower nighttime restfulness (HR, 0.910; p=0.009), and the acrophase (HR, 1.196; p=0.288). Depression was associated with RAR disruption (r=−0.338, p=0.041). Overall and somatic depressive symptoms were associated with activity phase shifts (morning to evening) (r=0.370, p=0.024).
Innominato et al., 2018 (94)	11 (5 M) advanced or metastatic colorectal or pancreatic cancer patients, median age 60	30-day actigraphy (Micro-Motionlogger)	Activity was prominent at daytime and restful at nighttime. Mean I<O values ranged from 96.3% to 98.5%. The average sleep efficiency was 81.9%–90.8% and <70% in approximately 1/10 of the nights. The average total sleep time was 8.6–9.7 h with an average midpoint of sleep of 01:02–05:30. I<O was independent predictive factor for all selected MDASI PROMs.
Innominato et al., 2018 (95)	Cohort 1: 237 metastatic colorectal cancer patients, not receiving anticancer Cohort 2: 31 advanced or metastatic gastrointestinal cancer patients	Cohort 1: 3-day actigraphy (Mini-Motionlogger) Cohort 2: 30-day actigraphy (Micro-Motionlogger)	The I<O was ≤97.5% in 54.9% of cohort 1 and 44.4% in cohort 2. A reduced I<O was significantly associated with increasing fatigue (p<0.0001), anorexia (p<0.0001), pain (p<0.0001), and sleep trouble (p<0.003). An increased I<O was significantly associated with greater values of global quality of life (p<0.0001), physical (p<0.0001), and social (p<0.0001) functioning but not role (p=0.02) functioning. Patients with a reduced I<O values had more interference with enjoyment of life, activity, relations with others and work (all p< 0.0001).
Chang and Lin, 2017 (96)	82 (59 M) stage 1–4 lung cancer patients, mean age 66.54	3-day actigraphy (Ambulatory Monitoring Inc.) and sleep diary	The I<O was 88.50, and higher I<O was associated with improved QoL (p<0.001).
Palesh et al., 2017 (22)	237 (148 M) metastatic colorectal cancer patients, median age 60.4	3-day actigraphy (Mini-Motionlogger)	Median values were a total sleep time of 7hr22, sleep efficiency of 90.56%, sleep latency of 8m40s, wake after sleep onset of 46m15s, I<O of 96.9%, bathyphase (lowest activity time) of 02:33, and average activity of 99 accelerations/minute. Patients with subjective sleep complaints had more circadian disruption (lower I<O) compared to those without sleep complaints (p=0.005). 40.1% of patients had subjective and circadian disruption (I<O <97.5%), 25.3% had subjective disruption alone, 14.8% had circadian disruption alone, and 19.8% had neither. Lowest health-related quality of life scores (including global, physical, social, and role functioning), and highest symptom scores (including fatigue and appetite loss), were seen in those with subjective and circadian disruption. Subjective difficulty sleeping was not associated with actigraphy sleep parameters.
Chen et al., 2016 (24)	Intervention group: 56 (24 M) stage 1–4 lung cancer patients, mean age 64.64 Standard care group: 55 (25 M) stage 1-4 lung cancer patients, mean age 62.51	3-day actigraphy (Micro-Mini actigraph) and sleep diary	Baseline (mean values): The r24 values were 0.42 (I) and 0.36 (C). A poor r24 was seen in 11/56 in the intervention group and 18/55 in the standard care group (26% overall). The I<O values were 94.68 (I) and 92.65 (C). A poor I<O was seen in 11/56 in the intervention group and 23/55 in the standard care group (30% overall). The total sleep time was 380.32 (I) and 395.06 (C). The sleep efficiency was 88.94 (I) and 88.36 (C). The sleep onset latency was 27.14 (I) and 31.85 (C). The wake after sleep onset was 45.86 (I) and 50.56 (C).
Ortiz-Tudela et al., 2016 (21)	24 (11 M), gastrointestinal cancer patients (21 metastatic), median age 63	4 and 8 days of wrist temperature activity and body position (accelerometery)	The L5 timing (centre of least 5 active hours) was 01.13 +/- 42 min. I<O before chemotherapy was 83.88%. Females had stronger and less fragmented rhythms than males. Significant gender differences were seen for interdaily stability (IS, a measure of rhythm stability) (p = 0.002), intradaily variability (IV, a measure of rhythm fragmentation) (p = 0.001), relative amplitude (RA, the difference between the mean of ten consecutive hours with the highest values and the mean of five consecutive hours with highest values divided by the combined value of both) (p = 0.001), circadian function index (CFI, combined IV/IS/RA) (p < 0.001) and I < O (p = 0.008).
Rebholz et al., 2018 (65)	57 (F) stage 0–5 breast cancer patients, mean age 52.32	3-day actigraphy (Ambulatory Monitoring Inc.) and 3-day salivary cortisol (waking, +30 min, 16:00, bedtime)	The r24 was 0.28 and wake after sleep onset was 0.15. African American females had more wake after sleep onset (p=0.017) and lower r24 (p=0.037) which persisted after adjusting for age and cancer stage. Wake after sleep onset and r24 were not significantly correlated with QoL.
Cash et al., 2015 (15)	43 (F) stage 0-4 breast cancer patients, mean age 52.49	3-day actigraphy (Micro Mini-motionlogger) and sleep diary and salivary cortisol (3 days—on waking, +30 min, 16:00, pre-bed)	The mean r24 was 0.27, nighttime restfulness (proportion of activity in bed that falls below the median out of bed activity) was 97.21%, daytime sedentariness (proportion of activity out of bed that is below the median in bed activity) was 6.59%, mean sleep time was 386.94 min, sleep efficiency was 89%, and wake after sleep onset was 13 min. Uncoordinated rest-activity rhythm, such as poor inter-daily stability, was associated with elevated markers VEGF, TGF-beta, and MMP-9 (associated with angiogenesis, immunosuppression, epithelial-mesenchymal transition, tumour invasion, and metastasis).
Dean et al., 2015 (97)	29 (18 M) stage 2a–4 non-small cell lung cancer patients, mean age 66.6	7-day actigraphy (Motionlogger) and sleep diary	Baseline actigraphy demonstrated poor sleep with 45% of patients sleeping <6 h The mean sleep efficiency was 77%, sleep latency was 51 min, and sleep duration was 5.9 h. Baseline sleep efficiency and wake after sleep onset were not associated with FACT-TOI, FACT-L, FACT-G, or the subscales. There were, however, associated after receiving chemotherapy.
Zeitzer et al., 2015 (16)	97 (F) recurrent or metastatic breast cancer patients, age 57.4	2-week actigraphy (Actiwatch 2) and sleep diary logs and Serum cortisol (20–60-min intervals from 3 h prior to bedtime to 1 h after wake time) Salivary cortisol 09:00, awakening and 30 min	No relevant findings from actigraphy
Chang and Lin, 2014 (98)	68 (34 M) stage 2–4 mixed cancer patients, median age 54.0	3-day actigraphy (Ambulatory Monitoring Inc.) and sleep diary	The median I<O was 89.5%. 34 patients were classified having a “disrupted” circadian rhythm (I<O ≤89.4) and 34 patients were classified as having a “regular” circadian rhythm (I<O ≥89.5) Survival was longer for patients with a “regular” circadian rhythm than a “disrupted” rhythm (3.9 months vs. 1.8 months, p<0.001, HR 2.19, p=0.006) Controlling for age, gender, cancer diagnosis, cancer stage, PSQI, Brief Pain Inventory-Chinese version (BPI-C), and Karnofsky Status (KPS) scale, a “dampened” circadian rhythm impacted survival (HR 4.59 p=0.001). Significant difference in cumulative patient survival rates were reported between “disrupted” and “regular” rhythm (p=0.005).
Levi et al., 2014 (99)	436 (273 M) colorectal cancer patients (427 metastatic), median age 59	2-7-day actigraphy (Mini-Motion logger)	The median I<O was 97.5% and a lower I<O was associated with poor WHO performance status (PS0 98.2%, PS1 96.5% PS1 and PS2/+ 91.5%, p<0.001). Women had a higher I<O than men (98.0% vs. 97.1%, p=0.04). Overall survival was longer in patients with an I<O >97.5% than those with an I<O ≤97.5% (21.6 months vs. 11.9 months, p<0.001). A 1% increase of I<O had a HR 0.954 (p=0.003). Progression free survival was also longer in patients with an I<O >97.5% than those with an I<O ≤ 97.5% (9.3 months vs. 5.8 months, p<0.001). Median I<O varied between patients without metastases (99.4%), with one metastatic site (97.4%), and with two or more metastatic sites (97.1%) (p=0.03).
Ma et al., 2014 (100)	68 (34 M) mixed advanced cancer patients, mean age 52.40	3-day actigraphy (Actigraph) and sleep diary	The median and mean r24 were 0.19 (−0.03–0.64). The median was I<O 89% and the mean I<O 85.29% (51%–100%). Patients spent a mean total time in bed of 461.19 min, mean total sleep time was 314.92 min, mean sleep efficiency was 75.95%, mean wake after sleep onset was 99.48 min, mean sleep onset latency was 40.65 min, and mean waking episodes was 12.66. Higher r24 was associated with longer total sleep time (r=0.26), improved sleep efficiency (r=0.29), shorter sleep onset latency (r=−0.24), and less wake after sleep onset (r=−0.28) (all p<0.05). Higher I<O was associated with longer total sleep time (r=0.38, <p<0.001) and shorter sleep onset latency (r=−0.49, p<0.01), improved sleep efficiency (r=0.30, p<0.05) and less wake after sleep onset (r=−0.25, p<0.05). r24 and I<O were negatively correlated with worst pain, pain intensity, and global PSQI score (all p<0.01). r24 predicted sleep quality and pain intensity (p<0.001).
Ortiz-Tudela et al., 2014 (101)	49 (25 M) mixed cancer patients (47 metastatic), median age 61.6	3–10-day actigraphy (Mini-Motionlogger)	A mean rhythmic 24-h pattern was observed for the whole group of patients. Circadian disruption during or after chemotherapy was significantly associated with developing clinically relevant fatigue (OR, 5.1; p=0.028), or body weight loss (OR, 6.1; p=0.05).
Palesh et al., 2014 (68)	97 (F) metastatic or locally advanced breast cancer patients, mean age 54.6	3-day actigraphy (Micro Mini-Motionlogger) and sleep diary and 2-day salivary cortisol (waking, +30 min, 12:00, 17:00, and 21:00)	Patients had mean total sleep time of 390 min, wake after sleep onset of 71.2 min, 14.5 wake episodes, and 4.8 min duration of wake episodes. The mean sleep efficiency was 84.6%, total time in bed of 478.3 min, and a sleep latency of 11.5 min. A higher sleep efficiency was associated with lower mortality (HR, 0.96; p<0.001). A 10% increase in sleep efficiency reduced subsequent mortality by 32%. The mean survival with a sleep efficiency of ≥85% was 68.9 ± 4.0 months compared with 33.2 ± 4.3 for a lower sleep efficiency (p<0.001). The sleep efficiency effect on survival remained after adjusting for age, oestrogen receptor status, treatments received, metastases, depression, and cortisol (HR, 0.94; p<0.001). There was no association between sleep duration and survival.
Dean et al., 2013 (102)	35 (34 M) stage 1-4 lung cancer patients, mean age 63.5	7-day actigraphy (Motionlogger) and sleep diary	77% patients had a sleep latency of more than 30 min and 61% slept <5 h per night. 88% of patients had a sleep efficiency of <85% and 91% were awake more than 30 min after sleep onset. Considering “good” and “poor” sleepers, actigraphy measures of sleep and measures of mood were not significantly different. Subjective and objective measures of sleep efficiency, sleep latency, sleep hours and wake after sleep onset were significantly different (all <0.05). There were no significant associations between the sleep diary and actigraphy variables of interest.
Dedert et al., 2012 (71)	57 (F) stage 1–4 breast cancer patients, mean age 52	3-day actigraphy and sleep diary and 3-day salivary cortisol (waking, 30 min after, 16:00, and bedtime)	The mean r24 was 0.27. Nighttime inactivity was 97.3%, and daytime inactivity was 6.1%. Intrusive thoughts were associated with a lower r24 and with daytime sedentariness. There was no association between intrusive thoughts and nighttime inactivity. More avoidant coping was associated with a lower r24 (p<0.05) and daytime inactivity (p<0.001). A higher autocorrelation co-efficient was significantly correlated with a steeper diurnal cortisol slope (p = 0.003).
Dhruva et al., 2012 (103)	73 (F) breast cancer patients (43.8% locally advanced), mean age 55.1	2-day actigraphy (Ambulatory Monitoring Inc.) and sleep diary	87% of patients had an “excessive” number of awakenings, 46% had abnormal wake after sleep onset, and 58% had total sleep times below healthy adult values. 25.6% had abnormal sleep onset latency and the mean value was 14.7. 46.2% had abnormal wake after sleep onset and the mean value was 11%. 87.2% had an abnormal number of awakenings with the mean number being 15.1. 57.7% had abnormal total sleep time with the mean of 419.8 min. 26.9% had abnormal sleep efficiency and the mean was 85.5%.
Innominato et al., 2012 (104)	77 (65 M) metastatic colorectal cancer patients, median age 62.3	At least 3-day actigraphy (Mini-Motionlogger)	The median and mean I<O were 97.5% and 95.1%. 39 patients (50.6%) had altered I<O of <97.5%. There was no significant association between I<O and progression free survival. Overall survival was associated with a more robust circadian rhythm (22.3 months vs. 14.7 months, p=0.013). This was independent of gender, treatment schedule, number of metastatic sites, rank of chemotherapy course of interest, and performance status on day 1 (p = 0.004). Patients with an altered rhythm during chemotherapy had a higher risk of earlier death (HR, 2.12; p=0.004). There were no significant differences between I<O and response rate or overall grade 3–4 toxicity rate. No clinical or biological parameters predicted the occurrence of a rhythm disturbance on treatment. Baseline disruption did not predict subsequent disruption, and there was no significant correlation between baseline and on-treatment I<O). There was no significant difference in toxicity in relation to circadian parameters.
Gibbins et al., 2009 (26)	60 (27 M) mixed advanced and incurable cancer patients, median age 67	7-day actigraphy (Actiwatch W-4) and sleep diary	Sleep efficiency was >90% for most patients with 12% of patients having a sleep efficiency <86%. Patients had 14–17 naps per day, lasting approximately 9 min each and 42%–48% of the day was spent immobile. Cancer diagnosis was not significantly related subjective or objective data. Subjective sleep quality was not correlated with objective sleep efficiency, sleep fragmentation, or daytime activity.
Innominato et al., 2009 (105)	130 (74 M) metastatic colorectal cancer patients, median age 60	At least 3-day actigraphy (Mini-Motionlogger)	The mean r24 was 0.38, and the median r24 was 0.37. The mean I<O was 94.3, and the median I<O was 97.0. The I<O and r24 were associated (r = 0.74, p<0.001) I<O and r24 were also associated with meanAct (mean activity levels) (p<0.001). There were no significant associations between progression-free survival and any CircAct parameter (I<O, r24, meanAct). Patients in the lowest I<O quartile (<92.4%) had the poorest survival (12.0 months), and patients in the highest I<O quartile (≥99.2%) had better survival (23.5 months). I<O and r24 were related to survival (HR, 0.95; p<0.0001 and HR, 0.20; p=0.004). Higher I<O, r24 and meanAct were associated with improved QoL and role functioning and less fatigue and appetite loss (all p≤0.01). Higher I<O and r24 were associated with improved social functioning, and less pain and dyspnoea (p≤0.01). Higher I<O and meanAct were associated with improved physical functioning (p≤0.01). Higher I<O was associated with less insomnia (p≤0.01). Actigraphy parameters were not correlated with emotional or cognitive functioning, nausea/vomiting, constipation, or diarrhoea. Patients with a good WHO PS had a more robust CircAct (I<O (p=0.01), r24 (p=0.0014), meanAct (p=0.047) that those with a poor PS. Lower I<O and r24 was associated with poorer WHO PS (PS0 I<O 98.2, r24 0.44, PS1 I<O 95.7, r24 0.36, PS2 I<O 95.3, r24 0.24). Use of analgesia was associated with a disturbed circadian rhythm (p<0.05 for all parameters). CircAct parameters did not correlated with age, gender, body mass index, number of metastases, site of primary tumour, blood tests, previous chemotherapy, or per cent of liver involvement, p>0.10). CircAct parameters did not predict for tumour response or toxicity.
Palesh et al., 2008 (75)	99 (F) metastatic breast cancer patients, mean age 54.65	3-day actigraphy (Micro Mini-Motionlogger) and 2-day salivary cortisol (waking, +30 min, 12:00, 17:00, 21:00)	Cancer patients had a time in bed of 478.5 min, sleep onset latency of 11.50 min, wake after sleep onset of 71.44 min, sleep efficiency of 84.5%, and 15 wake episodes. Longer time in bed was associated with lower pain intensity (r=−0.23, p=0.03), depression (r=0.21, p=0.05), younger age (r=−0.24, p=0.02), and chemotherapy (r=−0.29, p=0.006). Longer mean sleep latency was associated with lower perceived stress scale scores (r=−0.22, p=0.04) and the use of radiation (r=0.21, p=0.05). Mean sleep efficiency was associated with the dominant metastatic site being the chest alone vs. bone or viscera (r=0.27, p=0.01). Longer wake after sleep onset was negatively related to metastases to the chest only (r=−0.31, p=0.003) and positively correlated to hormone therapy (r=0.24, p=0.03). Higher mean number of wake episodes were associated with younger age (r=−0.23, p=0.03), metastases to the chest only (r=−0.23, p=0.03), sleep medication (r=0.26, p=0.012), and hormone therapy (r=0.23, p=0.03).
Rich et al., 2005 (78)	80 (52 M) metastatic colorectal cancer patients Group 1 (40) – “Good” circadian rhythm (r24 >0.47, <0.77), median age 59.6 Group 2 (40) – “Dampened” rhythm (r24 >0.03, <0.35), median age 60	3-day actigraphy (Actigraph) and 2-day serum cortisol (08:00 and 16:00)	“Good” circadian rhythm patients had a higher median r24 (0.58 vs. 0.22), I<O (98.45% vs. 91.36%), O>I (96.57% vs. 83.89%), and mean activity levels (107.5 vs. 84.0) than patients with “dampened” circadian rhythm (all p≤0.0001) “Good” circadian rhythm patients survived longer than “dampened” circadian rhythm patients (13.8 months vs. 11.1 months, p=0.0176). r24 and I<O were prognostic (p=0.001 and p<0.0001 in a univariate analysis). Patients with “Dampened” circadian rhythms had higher fatigue (60% vs. 40%, p=0.003), nausea/vomiting (33% vs. 10%, p=0.007), and appetite loss (50% vs. 28%, p=0.004) than patients with “good” circadian rhythms. “Good” circadian rhythm patients had higher QoL emotional and social functioning scores (p<0.0001). PS was worse in patients with “dampened” circadian rhythms (p<0.0008).
Mormont et al., 2002 (106)	192 (128 M) metastatic colorectal cancer patients, mean age 58	3–5-day actigraphy (Actigraph)	r24 was associated with I<O (r=0.67, p<0.001) and both r24 and I<O were associated with mean activity (r=0.38 and 0.21, p<0.01). I<O and r24 were highest in patients with a performance status 0 and lowest in those with a performance status 2. Higher I<O, r24 and mean activity was associated with less fatigue, appetite loss, and nausea/vomiting (all p≤0.05) Higher I<O was associated with less pain, constipation, and dyspnoea (all p≤0.002). Higher I<O and r24 were associated with less depression (all p≤0.01). Higher I<O and r24 were associated with improved global quality of life, physical functioning, social functioning, and emotional functioning (all p≤0.04).
Mormont et al., 2002 (49)	18 14(M) metastatic colorectal cancer patients, mean age 58	3-day actigraphy (Actigraph) and serum melatonin, 6-alphasulfatoxymelatonin and salivary cortisol Blood and saliva collected 3–6 h apart over 10–13 time points on day 1 and day 4	Actigraphy demonstrated a mean r24 of 0.37, median r24 of 0.41, mean I<O of 92.8 and median I<O of 94.2. 13 patients had an r24 >0.28, 10 patients had I<O >25% quartile Interindividual variation was noted in circadian rhythms of activity, hormonal, and haematological markers of circadian system function.
Mormont et al., 2000 (37)	192 (128 M) metastatic colorectal cancer patients, mean age 58	3-day actigraphy (Actigraph) and sleep diary % 2 days of blood cortisol (08:00 and 16:00)	The r24 ranged from -0.06 to 0.77 with a median 0.42. The dichotomy index (I<O) ranged from 49%-100% with a median of 97%. Patients with a higher r24 or I<O had longer survival (p<0.0001). Patients in the upper quartiles had a longer 2-year survival than those in the lower quartiles (38% vs. 8%). Higher I<O and r24 were associated with improved global quality of life and physical functioning, and lower depression scores (p≤0.01). Higher I<O, r24, and mean activity were associated with less fatigue and less appetite loss (p<0.001). Higher I<O was also associated with less pain (p=0.002). Higher mean cortisol was seen in patients with a low r24 (r=−0.17, p=0.04), or low I<O (r=−0.24, p=0.007). Cortisol rhythmicity was correlated with the r24 (r=0.16, p = 0.04). Self-rated sleep disturbances were not correlated to the rest–activity rhythm or mean activity levels. A poor performance status was associated with lower r24 and I<O (p<0.0001), and lower mean activity (p = 0.04). The probability of an objective response was significantly influenced by r24 (p=0.02) and I<O (p<0.0001).
Mormont et al., 1998 (50)	Study 1: 18 (14 M) metastatic colorectal cancer patients, age 35–72 Study 2: 109 (72 M) metastatic colorectal cancer patients, median age 59	Study 1: 3-day actigraphy and melatonin and cortisol (blood 9–11 time points, 6 hourly) Study 2: At least 3-day actigraphy activity	9/18 patients had a marked circadian activity rhythm. Wide interindividual variation was noted. High r24 (p=0.0005), dichotomy indices (p=0.0005) and activity levels (p=0.0002) were related to survival. Increases in r24 were correlated with less fatigue (r=−0.33, p=0.03), less appetite loss (r=−0.34, p=0.01), less depression (r=−0.24, p=0.04), improved global quality of life (r=0.29, p=0.009), improved social functioning (r=0.28, p=0.01), and performance status (p=0.008).

**Table 2D:** Polysomnography-related circadian rhythms and their associations in patients with advanced cancer.

Studies with a control group			
Article	Participants	Circadian measures	Outcomes relevant to review
Zeitzer et al., 2016 (52)	97 (F) recurrent or metastatic breast cancer, age 57.6 24 age-matched control subjects, age 57.1 (gender not identified)	28-h polysomnography and plasma cortisol (20–60-minute intervals) and 2-week actigraphy (Actiwatch 2) and sleep diary	Abnormal cortisol peaks were associated with polysomnography sleep disruption (p=0.004).
Silberfarb et al., 1993 (107)	Cancer patients: 17 (10 M) limited stage small-cell lung cancer or stage 2–4 non-small cell lung cancer, mean age 60.1 15 (F) stage 1–3 breast cancer, mean age 57.5 32 sex- and age-matched control subjects 32 patients with insomnia	3-day polysomnography and 2-week sleep diary	Cancer patients had longer total time in bed than any other group (vs. normal p<0.001, vs. patients with insomnia p<0.003). There was no difference in total time asleep between cancer groups (p=0.05) Sleep efficiency was lower in cancer patients (lung cancer vs. control subjects (p=0.007) and breast cancer vs. control subjects, (p=0.10)). Sleep latency was longer for lung cancer patients than control (p=0.03). Lung cancer patients had more difficulty remaining asleep than breast cancer patients (p=0.01) and control subjects (p=0.001).
Studies without a control group			
Padron et al., 2022 (91)	Cognitive behavioural therapy for insomnia and pain (CBT.i.p) group: 18 (F) stage 1–3 mixed gynaecological cancers, mean age 58.9 Psychoeducation (PE) group: 17 (F) stage 1–4 mixed gynaecological cancers, mean age 59.9	14-day polysomnography and actigraphy (Actiwatch-L) and sleep diary	Actigraphy and sleep diary data were not correlated. Baseline mean values (polysomnography vs. actigraphy) CBTi.p.: sleep efficiency was 75.5% vs. 80.8%, sleep onset latency was 24.7 min vs. 29.3 min, and wake after sleep onset was 91.1 min vs. 50.5 min PE: sleep efficiency was 71.0% vs 79.4%, sleep onset latency was 36.2 min vs. 33.6 min, and wake after sleep onset was 94.8 min vs. 53.4 min Objective (CBT.i.p and PE): 29% and 24% of patients had a SE >85% at baseline 76% and 50% of patients had SOL <30 min 6% and 13% of patients had WASO <30 min
Jakobsen et al., 2020 (29)	40 (24 M) mixed metastatic cancer diagnoses, median age 70	Overnight polysomnography and actigraphy (Actiwatch 2)	Results detail actigraphy findings and associations. Polysomnography findings not reported.
Parker et al., 2008 (108)	114 (58 M) stage 3–4 mixed cancer diagnoses, mean age 55.2	24-h polysomnography	Patients slept approximately 382 min/night (normal, 340–466 min) and 17.5% had <5 h sleep/night. Nocturnal sleep latency was <30 min (normal defined as <30 min), and sleep efficiency was 77.2% (normal defined as 86%–100%). >60 brief arousals occurred per hour and approximately 6 of the awakenings lasted at least 60 s/h. Patients slept in the daytime for an average of 89.3min. Total daytime sleep was associated with less nocturnal total sleep time (r_s_=−0.21, p=0.025) and less sleep efficiency (r_s_=−0.25, p=0.008), and more wake episodes (nocturnal index of awakenings) lasting at least 60 s (r_s_=0.27, p=0.003). Females had higher sleep efficiency than males (80.0% vs. 74.9%, p=0.042). White patients had more nocturnal total sleep time (409.8 min vs. 364.4 min, p=0.017) and higher sleep efficiency than non-white patients (80.2% vs. 75.3%, p=0.021). Patients living with another had greater nocturnal total sleep time (392.5 min vs. 336.9 min, p=0.035) and shorter sleep latency (23.5 min vs. 57.2 min, p=0.018) than patients living alone. A higher level of education was associated with a higher sleep efficiency (79.2% vs. 74.6%, p=0.013). Lung cancer patients had higher index of awakenings lasting at least 60 seconds than breast cancer patients (3.9 vs. 2.4, p=0.002). Total sleep time was increased in patients that use SSRIs (430.4 min vs. 372.7 min, p=0.009) and reduced in patients taking beta blockers (345.7 min vs. 388.5 min, p<0.047) Antineoplastics agents were associated with an increased sleep efficiency (83.4% vs. 76.1%, p=0.000). Beta blockers significantly decreased sleep efficiency (72.3% vs. 78.1%, p=0.047)

M, male; F, female.

### Investigative and reporting practice

Authors utilised actigraphy (n=34), cortisol (n=33), combined assessment methods (n=18), melatonin (n=11), and polysomnography (n=2) in their investigations.

Articles focused on different cancer diagnoses, including breast (n=24), gastrointestinal (n=22), mixed cancer diagnoses (n=22), lung (n=20), gynaecological (n=7), head and neck (n=2), and renal (n=1). All studies included patients with advanced or metastatic cancer; 40 studies focused solely on advanced or metastatic cancer patients.

Heterogeneity was seen in the investigational approach and the reported measures of circadian rhythm. Studies assessing melatonin used between 20 and 190 patients, sampled melatonin at 1–16-h intervals, and used between 2 and 10 different time points. Studies assessing cortisol used between 13 and 210 patients and sampled at 20-min to 12-h intervals. Sampling included fixed times, time slots, and/or were reported in relation to waking and bedtime. Melatonin and cortisol studies lasted between 24 h and 3 days for most studies.

Variation was also seen in the samples utilised. As an example, articles focusing on cortisol measures (n=30) used serum (n=14), saliva (n=16), serum and saliva (n=3), or urine (n=1) samples. Reported measures included descriptive statistics, mean levels (MESOR), variation between peak and trough levels at several time points (amplitude, double amplitude, 12- and 24-h amplitude), timing of peak level (acrophase), the area under the curve, the shape of changing levels between peak and trough levels (diurnal slope, phase angles), cortisol variability, and the change in cortisol levels on waking (cortisol awakening response, CAR).

Actigraphy studies included between 11 and 436 patients, with a duration of monitoring varying from an overnight study to 30 days. The number of reported actigraphy parameters were much larger, with overlapping characteristics. Across sleep and wake periods, the timing and duration of activities, the relative proportions between different activities, and the variability both within and between 24-h periods were considered. At least 50 different actigraphy parameters were reported. When normal actigraphy values were stated, they differed between studies. The dichotomy index (I<O), for example, which measures proportional in-bed activity to out of bed activity, was reported in 20 studies. Unfavourable, or disordered, I<O values ranged from <89.5% to ≤97.5% (17, 24, 28, 94, 95, 98, 99, 101, 104). Favourable, or regular, I<O values varied between ≥89.5% and ≥99% (8, 17, 22, 24, 28, 49, 94, 95, 98–101, 104).

Finally, polysomnography studies included between 35 and 121 patients, with the duration of monitoring varying from an overnight study to a 14-day study.

Many articles did not describe normal circadian patterns or values.

## Melatonin

### Melatonin patterns in advanced cancer

Significant interindividual variation in melatonin circadian rhythms was noted (9, 17). 24-h melatonin rhythms, with a peak-to-trough pattern, were noted in non-small cell lung, gastrointestinal, mixed gynaecological, and mixed cancer cohorts (9, 11, 17, 39, 40, 42, 43, 45, 46). Abnormal 24-h rhythms were noted in two studies and affected 17% of the patients with metastatic colorectal cancer and 100% of patients with small cell lung cancer (49, 51). Detailed abnormalities included a smaller evening melatonin rise and earlier dim-light melatonin onset (DLMO) for patients with a gastrointestinal cancer who demonstrate more in-bed to out-of-bed physical activity (lower I<O) (17). Smaller evening melatonin rises were also noted in patients with non-small cell lung, gastrointestinal, and cervical cancers, particularly in advanced stages (40, 41, 43, 46). Advanced breast and lung cancer patients had higher mean melatonin levels compared to early-stage disease or controls (47).

### Symptoms, quality of life, and survival

No statistically significant associations were reported between the melatonin circadian rhythm parameters and symptoms, quality of life, or survival in any of the included studies.

## Cortisol

### Cortisol patterns in advanced cancer

Normal and abnormal rhythms were noted across cancer cohorts to varying degrees. Ten studies reported abnormal cortisol circadian rhythms in cancer patients (9, 36, 49, 60, 61, 72, 76, 79, 82, 106). This included mixed cancer cohorts (100%), ovarian cancer (75%), breast cancer (60%), and colorectal cancer (28%–56%) (36, 49, 50, 72, 76, 79, 83). Four studies noted normal cortisol circadian rhythms, including in patients with gastrointestinal (“most”), non-small cell lung (100%), nasopharyngeal cancer, and breast cancer patients (17, 39, 52, 55). Where present, abnormalities included a flattened cortisol slope, or reduced diurnal variation, coinciding with a lower morning cortisol rise and/or an increased evening cortisol (9, 12, 13, 16, 42, 54, 55, 58, 61–64, 72, 76, 77). These changes, along with the area under the curve, were more pronounced with later stage, higher grade or metastatic disease, and poorer performance status (9, 12, 13, 18, 54, 61, 64, 66, 69, 74, 76, 82). Cortisol levels at separate time points were not significantly different between cancer stages, or when compared with controls, but the overall mean 24-h cortisol was higher in cancer patients (39, 42, 52, 74). The timing of peak cortisol level (acrophase) ranged from 04:38 to 09:30 (55, 59, 82).

The change from peak to trough (cortisol slope) was unrelated to education level, marital status, age, time since recurrence, PR status, and metastatic sites in a breast cancer cohort (67). Patients with progesterone-receptor-positive breast cancer did, however, have a smaller cortisol awakening response (77). A flatter cortisol slope was associated with being male (69).

Salivary and serum cortisol were positively correlated, particularly when a strong circadian rhythm was present (16, 80).

### Survival

Eight studies reported on survival, of which six report prognostic relevance of cortisol circadian rhythms. Patients with gynaecological cancer and either elevated night-time cortisol (HR, 1.802; p<0.001) or a flatter cortisol slope (HR, 1.633; p=0.001) had shorter survival (18). Conversely, patients with gynaecological cancer and more cortisol variability across the day had longer survival (HR, 0.644; p<0.001) (18). A lack of 24-h rhythmicity was prognostic in patients with lung cancer (HR, 68,052.8; p=0.009) and non-significantly in breast cancer (HR, 1.03; p=0.85) (68, 69). A flatter cortisol slope was prognostic in breast (HR, 464.9; p=0.0036) and renal cell cancer (HR, 1.88; p=0.002) (70, 81). Mean cortisol levels were prognostic in colorectal cancer (78). The early morning cortisol rise (CAR), area under the curve, and cortisol level at 00:00 were not prognostic in patients with lung cancer (61, 69). Altered cortisol rhythms in a study of patients with colorectal cancer were unrelated to survival (80).

Furthermore, a study of patients with breast cancer highlighted abnormal cortisol peaks, rather than the diurnal rhythm, to be associated with a shorter disease-free interval (r= −0.30, p=0.004) (52).

### Physical and psychological symptoms

A smaller cortisol awakening response, flatter cortisol slope, and less diurnal variability were associated with increased total symptom scores, individual scores for fatigue, and interference with general activity, work, and walking (54, 64).

Within lung, ovarian, and mixed cancer cohorts, reduced diurnal cortisol variation, elevated evening cortisol, elevated morning cortisol, and higher area under the curve were associated with depression (19, 53, 54, 73, 74). Patients with steeper cortisol slopes, and therefore healthier rhythms, expressed less negative affect during psychological therapy and demonstrated more post-traumatic psychological growth following diagnosis, and those with flatter cortisol slopes were found to repress emotions (14, 67, 79). Abnormalities, including higher waking cortisol and lower cortisol awakening response, were associated with antidepressant use in patients with breast cancer (77). Some studies reported no significant correlations between cortisol levels, or cortisol slope, and psychological measures (12, 70, 73).

### Quality of life

Flattened cortisol slopes, less cortisol variability, and elevated evening cortisol were associated with reduced physical well-being in patients with ovarian cancer (18, 54). Conversely, cortisol rhythms were also not correlated with quality-of-life measures for patients with breast cancer (65).

### Other

Abnormal cortisol peaks during sleep, coinciding with waking episodes, were reported in a subset of metastatic breast cancer patients (52). More frequent and longer lasting wake episodes and a progressive later waking time were also seen with flatter cortisol slopes (66, 75, 81).

The cortisol slope was correlated with the CAR, rather than waking level, and flatter slopes were associated with higher evening cortisol levels and an escape from cortisol suppression (63, 77).

## Actigraphy

### Actigraphy patterns in advanced cancer

Of the studies comparing patients with cancers to controls, 90% found abnormal actigraphy activity parameters in patients with cancer (17, 20, 23, 28, 30, 32, 85–88). Due to several reported parameters, the dichotomy index (I<O), 24-h autocorrelation coefficient (r24), and sleep efficiency (SE) are noted as examples.

Between 30%-95% of patients had a disrupted dichotomy index (I<O), suggesting proportionally more in-bed to out-of-bed activity, with average group values of 79%–98% (individual values of 49%–100%) (17, 21, 22, 24, 28, 37, 78, 87, 89, 94–96, 98–100, 104–106). Lower values suggest proportionally higher in-bed to out-of-bed activity. I<O values were lower in men, those with metastatic disease, and a poorer performance status (89, 99, 106). The I<O was reported to be the most discriminative parameter for cancer patients, although a large inter-subject variability was noted (17, 28).

Approximately 26%–28% of patients had a disordered 24-h autocorrelation coefficient (r24), representing the dissimilarity of rest–activity rhythms (RARs) between days, with average group values of 0.19–0.57 (individual values of −0.06–0.77) (15, 24, 32, 37, 49, 65, 71, 78, 88, 100, 105, 106). A r24 approaching 1 represents a prominent RAR (105). r24 values were lower in those with poorer performance status and in African-American women (37, 65, 105, 106).

Approximately 12%–88% of patients had disordered sleep efficiency, which measures sleep during time in bed, with average group values of 71%–92% (individual values, 20.2%–100%) (10, 15, 20, 22–27, 29, 30, 32, 68, 75, 85, 87, 91, 94, 97, 100, 102, 103). Higher values suggest that more time in bed has been spent sleeping, and lower values suggest that more time in bed has been spent awake.

Some studies reported circadian actigraphy parameters were unrelated to performance status, whilst others reported that time awake spent immobile (TASI), I<O, r24, and mean activity were significantly correlated with performance status (8, 17, 31, 37, 50, 78, 89, 99, 105, 106).

### Survival

A total of 12 studies commented on survival and all linked circadian disruption to survival. Stronger RARs evidence by improved dichotomy index (I<O), 24-h autocorrelation coefficient (r24), physical activity amplitude and MESOR, nighttime restfulness, sleep efficiency, and time awake spent immobile were associated with longer survival in patients with colorectal, breast, head and neck, non-small cell lung, and mixed cancer diagnoses (8, 9, 31, 37, 68, 78, 89, 93, 98, 99, 104, 105). In a mixed cancer cohort, disordered I<O was not prognostic; however, r24 and sleep efficiency were prognostic (89).

Examples of prognostic relevance include colorectal and mixed cancer cohort patients with an I<O <97.5%, or below median I<O, having a reduced overall survival (OS) of between 2.1 and 9.7 months, and reduced progression-free survival (PFS) of 4.2 months (98, 99, 104). Similarly, patients with colorectal cancer and an I<O ≥99.2% had 11.5 months longer survival than those with an I<O <92.4% (105). The I<O was an independent prognostic factor when accounting for factors including age, gender, performance status, cancer diagnosis and stage, previous chemotherapy, and surgery (98, 99, 104). Similarly, sleep efficiency was an independent risk factor for patients with breast cancer whereby those with a sleep efficiency >85% had over a double survival compared to those with poor SE (68). However, I<O, r24, mean activity, and sleep activity parameters were reported also reported to not be significantly correlated with overall survival or progression-free survival (68, 89, 92, 104, 105).

### Physical and psychological symptomatology

Abnormal circadian activity rhythms were associated with pain, fatigue, drowsiness, nausea, vomiting, anorexia, and weight loss (20, 27, 37, 50, 78, 101, 106). Higher I<O values were specifically associated with less pain, fatigue, anorexia, sleep disturbance, constipation, and dyspnoea, and improved sleep quality (95, 100, 105, 106). Higher r24 values were specifically associated with less insomnia, daytime dysfunction, fatigue, anorexia, pain, and dyspnoea (20, 30, 100, 105). Higher sleep efficiency was associated with less pain (92). Greater time to sleep once in bed (sleep onset latency, SOL), wake after sleep onset (WASO), and time in bed (TIB) were associated with gastrointestinal symptoms in a mixed cancer cohort (92). Increase time spent napping was associated with increased pain, fatigue, and daytime sleepiness (92). No association between circadian activity parameters and pain or fatigue was found in a mixed cancer cohort (8).

Lower sleep efficiency, I<O, r24, and mean activity along with increased time spent napping or in bed were all associated with increased depression (37, 50, 75, 92, 93, 106). A lower r24 and more daytime inactivity were associated with intrusive thoughts and avoidant coping in patients with breast cancer (71). Studies also reported that anxiety and depression were not associated with sleep–activity rhythms, including the I<O (8, 17, 23).

### Quality of life

Circadian disruption was associated with interference with activity, work, relations, and enjoyment of life for patients with colorectal cancer (95). Improved r24, I<O, and meanAct were associated with improved global QoL, along with health, physical, social, and functioning subscores (20, 50, 78, 95, 96, 105, 106). A lower amplitude and MESOR and a later acrophase were associated with worse global QoL in a mixed cancer cohort (8). The strongest correlation between an actigraphy parameter and quality of life measure in a mixed cancer cohort was the 24-h correlation coefficient (32). Studies also noted that circadian parameters were not associated with the fatigue, emotional, or cognitive subscales of quality-of-life measures (105, 106). One study of patients with breast cancer noted that WASO and r24 were unrelated to global QoL (65).

### Other

There were mixed reports regarding chemotherapy response and circadian rhythmicity in patients with colorectal cancer. One study noted that disordered rhythmicity during chemotherapy was associated with earlier death but not to objective response or toxicity, while another study noted objective response to be influenced by r24 and I<O (37, 104). Patients receiving chemotherapy who also had evidence of circadian disruption were more likely to experience weight loss and fatigue (101).

I<O appeared to correlate with circadian temperature rhythms, self-reported physical activity, and chronotype (17). More robust circadian rhythms were associated with greater light exposure (8).

Subjective and objective measures differed for physical activity but were closely correlated for sleep (27, 102). Subjective sleep disruption and circadian disruption can occur together or independently (22). Although total sleep time (TST), SE, and WASO were associated with subjective sleep quality, physical activity measures were also not significantly different between those who report their sleep as good or poor (26, 29, 37, 85, 102).

Subjective scores of pain and physical function correlated with objective physical activity, and those using analgesia had more abnormal circadian activity rhythms (84, 105). Daytime sleep, or inactivity, was related to sleep medication use, night-time sleep disturbance, daytime dysfunction, night-time sleep, and sleep quality (30, 38, 108). Sleep efficiency was reported to be correlated with chest metastases, hormone use, and radiotherapy (75). Patients with a higher r24 had less daytime dysfunction and less insomnia (20, 30). Circadian disruption was associated with tumour progression markers (15).

### Polysomnography

Patients with cancer spent more time in bed were noted to have multiple nocturnal awakenings and had an average sleep efficiency of up to 77.2% (91, 107, 108). Increased daytime sleep was associated with less night-time sleep and more nocturnal awakenings in a mixed cancer cohort (108). Medications were also found to impact on sleep. Anticancer therapies were associated with increased sleep efficiency, whereas beta blocker use was associated with reduced sleep efficiency (108). Sleep efficiency was higher in women, white patients, and those with a higher education level (108).

### Correlations between measures of circadian rhythm

Increased diurnal physical activity variability was associated with increased diurnal melatonin and cortisol variability along with an earlier DLMO (17). Salivary cortisol levels appeared unrelated to I<O, cortisol rhythmicity positively was correlated with r24, and more robust actigraphy rhythms were associated with a steeper cortisol slope (17, 37, 71).

The dichotomy index was correlated with r24, mean activity, sleep motor activity, sleep efficiency, and WASO (28, 89). r24, I<O, and mean activity were also correlated (106).

Higher I<O and r24 were associated with improved sleep efficiency (100).

Polysomnography-derived values for sleep efficiency were lower, and wake after sleep onset higher, than actigraphy-derived values (91).

## Discussion

This review supports, expands upon, and updates several previous reviews of circadian rhythmicity in patients with advanced cancer. It highlights that, for several patients with advanced cancer, disordered cortisol, melatonin, and physical activity circadian rhythms are associated with increased symptom burden, poorer quality of life, and shortened survival. Other important associations with CRDs include poorer performance status and raised biomarkers of tumour progression.

A review of rest–activity rhythms in advanced cancer patients found that CRDs are particularly evident amongst men, those undergoing chemotherapy, and those who were symptomatic (109). Additionally, circadian disruption may be seen across the cancer trajectory, with worse biopsychosocial outcomes reported in cancer survivors who have disordered cortisol rhythms (110). This review highlights that circadian rhythms may be maintained in some patients with cancer and that wide inter-individual variation exists. Future research aimed at identifying patients that are at risk of circadian rhythm disorders, and impacted by their associations, is important, particularly when considering interventional studies to improve circadian rhythms and patient outcomes.

Articles were predominantly observational in nature, and many studies lacked a control group. Causality is difficult to establish, particularly due to the bi-directional relationship between cancers and circadian rhythm disorders, and the influence from external factors. CRDs impact on several neuroendocrine-immune functions, including inflammatory responses and hormonal secretion, and predispose individuals to developing cancer (111). Cancer in turn generates a pro-inflammatory state, and increased circulating cytokines levels can disrupt circadian rhythms (111). Rest–activity patterns are influenced by age, sex, race, education, and voluntary behaviour (6, 112). Cortisol values are influenced by sex, age, body mass index, menstrual cycle, sleep disturbances, renal disease, and acute illness, for example (4). The review highlights studies of patients prior to, during, and after anticancer therapies, and within the inpatient and community setting. Limited information on previous and current therapeutic regimes, and location of metastatic disease, limits the ability to synthesise findings. Potential modifying factors of circadian rhythmicity should be reported and taken into account when reviewing findings (113).

CRDs and their impact are not solely seen in cancer patients. Circadian disruption has been reported in patients with neurodegenerative conditions including Alzheimer’s disease, Parkinson’s disease, and Huntington’s disease (114). Despite conflicting findings, evidence highlights altered rest–activity, body temperature, melatonin, and cortisol rhythms within this population and associations with physical and psychological well-being, and quality of life (114). At present, the similarities in circadian disruption between clinical conditions are not clear.

Furthermore, the review highlights heterogeneity in the investigation and reporting of circadian rhythms in cancer patients and a lack of threshold values to identify circadian parameter abnormalities. Several reporting measures, overlapping definitions, and an absence of clear definitions were found in the investigation of circadian rhythms, particularly when using actigraphy. Heterogeneity in actigraphy research is not limited to cancer populations. A review of 126 actigraphy studies of children highlighted a lack of standardisation in actigraphy practice, including the reporting of epoch length, artefact detection, and definition of variables (115). Additionally, within cohorts of bipolar disorder patients, over 30 possible actigraphy parametric and non-parametric measures were reported (116). In this review, only four actigraphy parameters (I<O, r24, mean activity, and SE) were associated with at least three of the areas of interest (physical symptoms, psychological symptoms, quality of life measures, and survival). Although a wealth of information can be obtained using actigraphy, the reporting parameters should be aligned with the overall study objectives to allow a clear message in the literature. Analysis of actigraphy data takes many forms and lacks standardisation (6, 112). Similarly, variable sampling protocols, analysis, and reporting practices has been seen in cortisol and melatonin studies (3, 110). When faced with such heterogeneity in approaches, it is challenging to make firm conclusions, and standardisation may improve research practice. The development of recommendations to identify, and subsequently report, optimal sampling processes, particularly the frequency and timing of samples, and the calculation of circadian parameters are required.

Actigraphy data can report the timing of events, duration of events, or relationship between events. Although studies may focus on “sleep–wake” or “rest–activity” periods, there is significant overlap. Diagnostic criteria have been formulated for circadian rhythm sleep–wake disorders by American Academy of Sleep Medicine (117). The diagnosis considers the timing of sleep onset and offset, and the presence of jet lag or shift work, to categorise patients into seven different diagnoses. Many studies of advanced cancer patients reported actigraphy measures across the 24-h period rather than focusing on this timing of sleep onset–offset. The circadian activity rhythm disorders in cancer patients are likely separate to intrinsic circadian sleep–wake rhythm disorders. Recent international consensus recommendations have been developed for the assessment and diagnosis of circadian rest–activity rhythm disorders (CARDs) (118). The recommendations outline key modifiers of circadian rhythmicity, areas to consider within a clinical history, patient sleep and activity diary, and accelerometery during assessment, and criteria to diagnose a CARD. Diagnostic criteria of other forms of CRDs do not currently exist.

The scoping review was strengthened by using independent authors at multiple stages of the review process. Additional evidence was actively sought through hand searching review papers and reference lists. The scoping review is inclusive of available evidence and placed minimal limitations in the search strategy. It made no attempt to critically analyse the quality of evidence. Although the review aimed to focus on advanced cancer patients, several studies included non-advanced cancer patients. This approach may dampen associations, but it was felt to be more inclusive and to provide a broader insight of the topic. Furthermore, the review did not exclude several confounding factors in selected articles, such as medications and chemotherapy. This information was not available in several studies, and through exclusion, it would have limited the generalisability of the findings. Studies reporting on circadian rhythmicity in patients with cancer would benefit from detailed information on recent and existing modifiers of circadian rhythmicity, and the presence and location of metastatic disease.

**Box 1:**

**Gaps in the current literature**.

■ What are the risk factors for a patient with cancer to develop a circadian rhythm disorder?

■ How do circadian rhythm disorders differ between malignant and non-malignant clinical conditions?

■ How do circadian rhythm disorders different between malignant subgroups?

■ What are the optimal measurement and analytical approaches when assessing cortisol, melatonin, and rest–activity circadian rhythms?

■ What are the abnormal threshold values for cortisol, melatonin, and rest–activity parameters when diagnosing a circadian rhythm disorder?

■ Do current investigative approaches translate into the clinical setting, considering the ease and acceptability for patients and clinicians?

## Conclusion

Cancer patients, particularly those with advanced disease, are at risk of circadian rhythm disorders and significant associated complications. It remains unclear which subset of patients are most susceptible. Conflicting results within the review highlight the need for further studies to identify patient populations that are most impacted by circadian rhythm disorders. Current investigative approaches require a multiple sampling approach (blood, urine, and saliva) or a prolonged period of activity monitoring. In the clinical setting, and advanced cancer population, this may require an alternative approach. Current gaps in the literature are highlighted in **Box 1**. There needs to be an attempt to standardise research approaches and reporting practice within circadian rhythm research and to develop criteria to identify circadian rhythm disorders. Research standardisation and targeted approaches may help in future research aimed at developing management approaches to circadian rhythm disorders.

## Author contributions

CG was responsible for the conceptualisation of the review and development of the search strategy. CG and JP conducted the scoping review, data extraction, and data checking. CG wrote the initial draft of the manuscript with editorial input from JP and AD. All authors contributed to the article and approved the submitted version.
